# On the Origin of Biomolecular Networks

**DOI:** 10.3389/fgene.2019.00240

**Published:** 2019-04-10

**Authors:** Heeralal Janwa, Steven E. Massey, Julian Velev, Bud Mishra

**Affiliations:** ^1^Department of Mathematics, University of Puerto Rico, San Juan, PR, United States; ^2^Department of Biology, University of Puerto Rico, San Juan, PR, United States; ^3^Department of Physics, University of Puerto Rico, San Juan, PR, United States; ^4^Departments of Computer Science, Mathematics and Cell Biology, Courant Institute and NYU School of Medicine, New York University, New York City, NY, United States

**Keywords:** biomolecules, regulation and communication, interaction (binary) relationship, network model, network analysis, spectral analysis

## Abstract

Biomolecular networks have already found great utility in characterizing complex biological systems arising from pairwise interactions amongst biomolecules. Here, we explore the important and hitherto neglected role of information asymmetry in the genesis and evolution of such pairwise biomolecular interactions. Information asymmetry between sender and receiver genes is identified as a key feature distinguishing early biochemical reactions from abiotic chemistry, and a driver of network topology as biomolecular systems become more complex. In this context, we review how graph theoretical approaches can be applied not only for a better understanding of various proximate (mechanistic) relations, but also, ultimate (evolutionary) structures encoded in such networks from among all types of variations they induce. Among many possible variations, we emphasize particularly the essential role of gene duplication in terms of *signaling game theory*, whereby sender and receiver gene players accrue benefit from gene duplication, leading to a preferential attachment mode of network growth. The study of the resulting dynamics suggests many mathematical/computational problems, the majority of which are intractable yet yield to efficient approximation algorithms, when studied through an algebraic graph theoretic lens. We relegate for future work the role of other possible generalizations, additionally involving horizontal gene transfer, sexual recombination, endo-symbiosis, etc., which enrich the underlying graph theory even further.

## 1. Genesis of Bio-molecular Interactions

### 1.1. Introduction and a Road Map

A range of complex phenotypes of biomolecular systems can be inferred from macromolecular interactions, represented using combinatorial networks. Such biomolecular networks include gene (regulatory) networks (GRNs) (Thompson et al., 2015), protein-protein interaction (PPI) networks (Huang et al., 2017), protein and RNA neutral networks (Schuster et al., 1994; Govindarajan and Goldstein, 1997), metabolic networks (McCloskey et al., 2013), and meta-metabolic networks (composite metabolic networks of communities) (Yamada et al., 2011). Here, we will focus on the neglected role of information asymmetry between genes and their gene products, which is identified as a key factor distinguishing biochemistry from abiotic chemistry in early life, and which has subsequently influenced biochemical processes. Such pairwise interactions led to the establishment of the earliest biomolecular networks, and their nature influenced subsequent network growth. We will concentrate on GRNs and PPI networks as illustrative examples, but the principles outlined are also applicable to the other types of biomolecular networks. We focus on mathematical and algorithmic techniques that by analyzing evolutionary dynamics may shed light on possible approaches to speculate on the very “origin of networks,” and the challenges they pose. For simplicity, we illustrate the approaches highlighting “Evolution by Duplication” (EBD); other dynamics may be handled *mutatis mutandis*.

The paper will adhere to the following road-map, aimed at identifying and explaining several challenges for the field of “evolutionary biology of networks” by first building on a review of biological and mathematical notions and frameworks, within which the open questions are formulated. First, a brief introduction presents biomolecular networks, and biomolecular signaling games between gene players. Second, it is followed by a consideration of the role of gene duplication from the perspective of information asymmetry. Switching to a mathematical formulation, a compendium of known results in (algebraic and combinatorial) graph theory are presented, comprising a toolbox for addressing the topics raised here. Last but not least, a series of open problems are described. These open problems focus largely on the following: How to devise efficient (algebraic) algorithms that can shed important lights on *game theoretic models of the evolution of biomolecular interactions*, given that they are driven by information asymmetry (leading to duplications, complementation, pseudogenization, etc.). Some of these important mechanisms have been studied qualitatively elsewhere, albeit not mathematically rigorously.

### 1.2. Ohno's Evolution by Duplication

At the genetic level, the growth of a GRN (gene regulatory) or PPI (protein-protein interaction) network is driven by gene mutation, including duplication, translocation, inversion, deletion, short indels, and point mutations, of which duplication plays an outsized role, although as we incorporate other known and unknown mechanisms (e.g., non-orthologous gene displacement, HGT, sexual recombination, etc.) a more complete picture may emerge. Susumu Ohno coined the phrase “evolution by duplication” (EBD) to emphasize duplication in the evolutionary dynamic (Ohno, 1970). Consequently, we will mainly consider the process of gene duplication, but the principles outlined may be regarded as an idealization, which may be extended to other mutational processes—some yet to be discovered.

The classic view of molecular evolution is that gene families may expand and contract over evolutionary time largely due to gene duplication and deletion (Demuth et al., 2018). Here, we wish to present a more complex view, by exploring how biomolecular networks may grow, contract, or alter their topology over time, from the relative dynamic contributions and interactions of their constituent genes and gene families, and we do so through the prism of signaling game theory. Mechanistically, this evolution is driven in large part by the process of gene duplication and deletion, which lead to node and edge addition, or removal, from a biomolecular network, respectively. Since such variations in the network alter the phenotypes, over which selection operates, the evolution of networks and their features ultimately capture the essence of Darwinian evolution.

Recently, we introduced a signaling games perspective of biochemistry and molecular evolution (Massey and Mishra, 2018). There, we focused on interactions between biological macromolecules, which may be described using the framework of sender-receiver signaling games, where an expressed macro-molecule such as a protein or RNA, constitutes a signal sent on behalf of a sender agent (e.g., gene). The signal comprises the three-dimensional (3D) conformation and physico-chemical properties of the macromolecule. A receiver agent (e.g., a gene product, another macromolecule) may then bind to the signal macro-molecule, which produces an action (such as an enzymatic reaction). The action produces utility for the participating agents, sender and receiver, and thereby—albeit indirectly—a change in overall fitness of the genome (in evolutionary game theory, utility and fitness are treated as analogous). When there is common interest, the utility is expected to benefit both sender and receiver and their selection, thus driving Darwinian evolution.

Replicator dynamics allow the signaling game to be couched in evolutionary terms (Taylor and Jonker, 1978). These arise from the increased replication of players with higher utility (fitness). Thus, if a gene has a strategy that results in increased utility, then it will increase in frequency in a population. For a sender gene this would entail sending a signal that results in an increase in utility, while for a receiver gene this would entail undertaking an action that likewise results in an increase in utility. As already suggested, these dynamics represent a process analogous to Darwinian (adaptive) evolution or positive selection.

**Figure 1:**
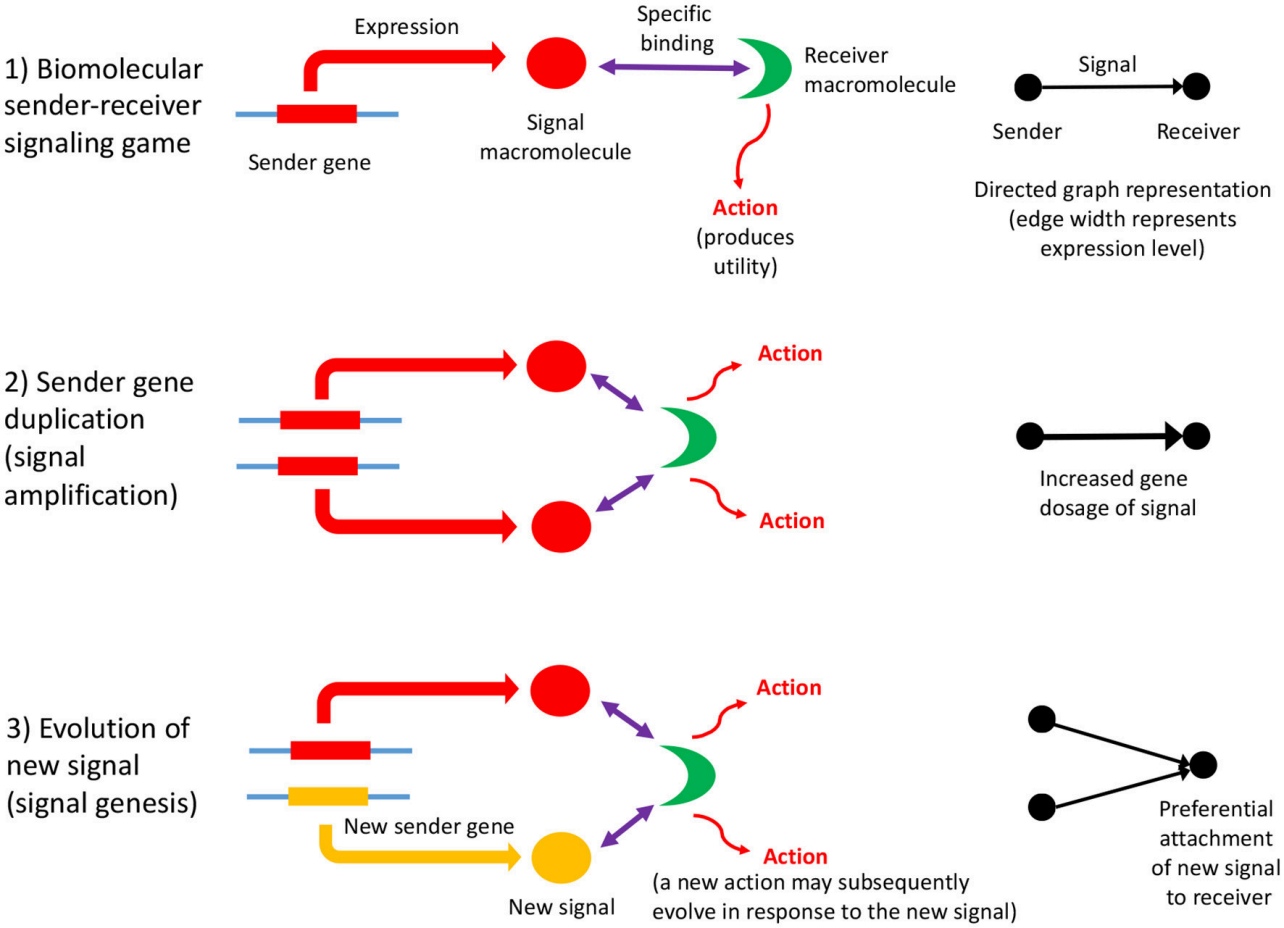
The influence of information asymmetry on growth of a PPI network. Interactions between macromolecules are envisaged as a biomolecular signaling game whereby a sender gene expresses a macromolecule, the signal, that then binds specifically to a receiver macromolecule, which then undergoes an action (such as an enzymatic reaction, or conformational change), which produces utility (fitness). The signal consists of the three-dimensional conformation and physicochemical properties of the macromolecule (1). The sender gene may undergo duplication, which has a dosage effect on the expressed macromolecule, resulting in signal amplification (2). This mechanism is expected to lower the Shapley value of the gene players in the genome, as the signal is partially redundant and so inefficient. Subsequently, the sender gene duplicate may acquire a new function (evolve a new signal) although the majority would be expected to undergo pseudogenization (3). Both these scenarios represent the re-establishment of a Nash equilibrium. If a new signal macromolecule evolves, it is likely to bind to the same receiver macromolecule initially. This preferential attachment arises because gene duplicates have a tendency to bind to their original interaction partner initially, and then subsequently undergo interaction turnover (Zhang et al., 2005), and is illustrated to the right of the figure. A key problem is how a new action by the receiver arises as the result of the evolution of a new signal; the new action may co-evolve with the new signal, or may be necessary first before a new signal can evolve. The latter would imply that receiver gene duplication and action genesis facilitates the evolution of new signals and sender genes (an exception would be when there is a conflict of interest; here the sender is more likely to make the first move in evolving a novel deceptive signal, and then the receiver would respond with a better discriminative recognition mechanism). This key, and novel aspect of gene duplication might be deciphered via consideration of the topology of directed graph representations of biomolecular interactions as sender-receiver signaling games. Refinements to the illustrated scheme include situations where the original signal protein binds to a variety of receiver proteins, or where the gene that codes for the receiver protein undergoes duplication (Figure 2).

Biomolecular signaling games are sustained by information asymmetry between sender and receiver, and so their interactions can be represented using directed graphs (as defined in section 2). Information asymmetry arises because the receiver is uninformed regarding the identity of the sender gene: it must rely on the signal macromolecule to determine its identity. But, this strategy may be open to deception. However, most biomolecular signaling games in the cell are between sender and receiver genes which have perfect common interest. This is so, because they are *cellularized*, chromosome replication is synchronized and so the genes replicate in concert. Such games are termed “Lewis signaling games,” and rely on honest signaling from sender to the receiver, which constitutes a signaling convention (Lewis, 1969). A biomolecular signaling game is illustrated in Figure 1, part (1).

On occasion, situations may arise where a sender has a conflict of interest with the receiver. In the cell, this kind of misalignment of interests can occur when a sender gene is selfish, and would prefer to replicate itself at the expense of the rest of the genome. Such genes are termed “selfish elements,” and come in a variety of forms, marked by decoupled replication from the rest of the genome (Burt and Trivers, 2006). In a signaling game, when there is such a conflict of interest, then the sender is expected to adopt a degree of deceptive signaling (Crawford and Sobel, 1982). Consistent with this, there are a range of selfish elements that utilize molecular deception, which implies that there is a cost to the host genome (Massey and Mishra, 2018). In addition, cancer and pathogens also make widespread use of deceptive strategies at the molecular level, which is expected given their clearly opposed interests with the host (Massey and Mishra, 2018).

The importance of information asymmetry at the molecular level is manifold. Given that information asymmetry leads to the possibility of molecular deception, this means that in a biomolecular network, in principle honest and deceptive signals could be mapped as honest or deceptive biomolecular interactions, respectively. This viewpoint may have importance in better understanding of processes such as cancer, in which molecular deception plays a central role in its progression (section 4.3) where we also formulate open problem 4.H), as well as of the dynamics of persistent infections. Given the harmful effects of molecular deception, it is necessary to reduce information asymmetry, in order to promote cooperation between gene players, in the normal functioning of the genome. For instance, in the theory of incomplete contracts, a topic linked with the economics of information, reduction in information asymmetry reduces the likelihood of deception between parties, which consequently promotes trust (Devos et al., 2012) and so cooperation (Lorenz, 1999). Given this framework, one may suggest a form of “molecular trust” that is promoted when the information asymmetry between two gene players is reduced (effectively increasing transparency), with the effect of promoting utility (fitness) for both players, since deception is less likely to occur. One means to achieve this effect is by the use of costly signals, which are costly to produce and so are more likely to be honest (Veblen, 1899; Spence, 1973; Zahavi, 1975); such signaling establishes “molecular trust” because mimicking the signals is expensive. In biomolecular terms, a costly signal is represented by the unique 3D conformation and physico-chemical properties of a macromolecule, which are difficult to imitate given its complexity.

When biomolecules are expressed from sender genes of an unknown type, identity signals are necessary, and so information asymmetry provides additional explanatory power for understanding the dynamics of molecular recognition. Biomolecules may be considered as belonging to two groups, namely, self and non-self, corresponding to cooperative members of the genome, or not, respectively. Self or non-self biomolecules might be equivalated to an in-group and out-group respectively, in sociological terms, and this view then might then imply some loose parallels between the dynamics of bio-molecular and social networks. In this context, it is of interest to consider how non-self gene players may become integrated into the cell and its biomolecular networks. This process may result from the endosymbiosis of a microbial genome, or the acquisition of plasmids. As the non-self genes evolve increasing cooperativity with the host genome over time, the occurrence of molecular deception is expected to reduce. This is because the level of deception is correlated with the level of conflict of interest (Martinez and Godfrey-Smith, 2016) with the host genome; the greater the misalignment of interests, the greater the level of deception that is expected from the non-self genes (Massey and Mishra, 2018).

Under the scenarios supported by a game theoretic framework, one may speculate how biomolecular networks may have originated. The very first biomolecular interactions in early life would have been characterized by molecular specificity, a distinguishing feature of biochemistry (Konnyu and Czaran, 2011). Molecular specificity arises when organic molecules reach a certain size, additional size being necessary to bind a smaller ligand. Molecular specificity is a form of recognition, which effectively allows verification of a ligand. Considering the ligand as a signal, then the macromolecule is the receiver, and the gene that produces the ligand can be considered the sender agent. The very first biomolecular network would have consisted of two nodes, sender and receiver, with the edge connecting the two representing the signal. As more biomolecular interactions evolved, the network increased in numbers of nodes and edges. Increases in organismal complexity may be viewed as an increase in the numbers of genes in the genome, but the numbers of biomolecular interactions has more explanatory power. Thus fully understanding the nature of these interactions and how they evolve is necessary for better understanding the emergent phenotype of an organism. In the genome of the ancestral life form, once a number of genes with separate function had evolved, it then would have become beneficial to evolve gene regulation. Therefore, genes with the dedicated function of regulating other genes in the genome would have arisen (transcription factors). The combination of regulatory and functional genes would have comprised the first GRN. Increases in organismal complexity have been facilitated by an increase in the complexity of the GRN (Burton, 2014).

Gene duplication, accompanied by the establishment of new biomolecular interactions, therefore is a fundamental evolutionary driver of organismal complexity (Lespinet et al., 2002), from the first life forms onward. Although the precise mechanism(s) of gene duplication remains to be established (Reams and Roth, 2015), some generalities may be made in terms of signaling games. The first step in the process of duplication of a sender gene may be viewed as one of signal enhancement. Because gene duplication results in gene dosage effects, it also results in amplification of the signal, the expressed gene product (resulting in weighted graphs—discussed in section 2). This strategy can be viewed as lowering the overall utility of the genome, given that there is a cost involved in producing excessive signal. It is, thus, expected to lower the Shapley value (Shapley, 1969) of the gene players that cooperate within the genome. This conflict is usually resolved when the duplicated gene becomes pseudogenized, the usual fate of gene duplicates (Innan and Kondrashov, 2010).

Subsequent to duplication, the gene duplicates will sometimes diverge in function, although the exact mechanism remains to be elucidated (Innan and Kondrashov, 2010). This process represents signal divergence if the gene is a sender gene, and action divergence if the gene codes for a receiver macromolecule. The genesis of a new sender gene with a new signal may then promote evolution of a novel action by the receiver macromolecule, potentially facilitating duplication of the receiver gene itself. Likewise, the duplication of a receiver gene may facilitate the diversification of macromolecular signals that interact with the two duplicated receiver macromolecules. The process modifies the GRN or PPI network in a non-obvious manner and it deviates considerably from the way evolution of random graphs is usually treated, following Erdös and Rényi, discussed in more detail in section 3 (Erdös and Rényi, 1959). These entail more complex random network evolutionary models (several of which are discussed in further detail in section 3).

Signal and action genesis via gene duplication may have features in common with a Pólya's urn model of signal genesis (Alexander et al., 2012) (Pólya's urn models are statistical models that involve sampling with replacement influenced by the identity of the sampled element. These models can lead to a “*rich get richer*” effect, of which “preferential attachment” is an example, discussed in more detail in subsection 3.2). In this model, reinforcement of signals (similar to reinforcement learning) may promote the invention of new synonyms. These considerations may provide parallels for how signals originate elsewhere, not dissimilar to how new words in a language can arise from existing words by a process of derivation (Cotterell et al., 2017). Mechanistic commonalities in the process of signal genesis in these diverse systems as exhibited in GRNs remain to be explored. These models hint at a possibly new, but universal form of “preferential attachment” that drives the variations in biomolecular networks as well as the selectivity in Darwinian evolution.

### 1.3. Network Topology, Evolution by Duplication, and Preferential Attachments

Consequently, the topology of gene networks is non-deterministic and yet not memoryless, since it must encode layers of ripples produced earlier via the dynamics of gene duplication (paralogs and orthologs), as amplified during the network's history. Just as physicists infer the theories of origin of the universe from the cosmic background radiation, we expect to enrich our understanding of the origin of machinery of life (e.g., codon evolution, evolution of multicellularity, evolution of sex etc.) from a rigorous analysis of the signaling games and their equilibria, which has rippled through the extant biomolecular networks. Taking this analogy further, we observe that the ripples in gravitational waves have been proposed to reflect the existence of parallel universes, whose presence created asymmetries in the initial conditions, giving rise to filamentary structures in the visible universe (Hawking and Hertog, 2018) This comparison is inspired by the notion of a “protein big bang” from a single (or handful of) ur-protein(s) in the first complex life forms, evolving by gene duplication into the extant “protein universe,” hinting at the information asymmetries fossilized in the GRN and PPI networks (Dokholyan et al., 2002).

Likewise, we point out that information asymmetry in macromolecular sender-receiver interactions may point to evolutionary paths that might have been abandoned unexplored; which may suggest new engineering approaches needed by synthetic biology, or in drug discovery, or immuno-therapy. Note that during the process of evolution of signaling, gene duplication and deletion contribute to a certain degree of non-determinism and “conventionality” to the Nash equilibria that stabilize and manifest as non-trivial anisotropies in gene network topology.

**Figure 2:**
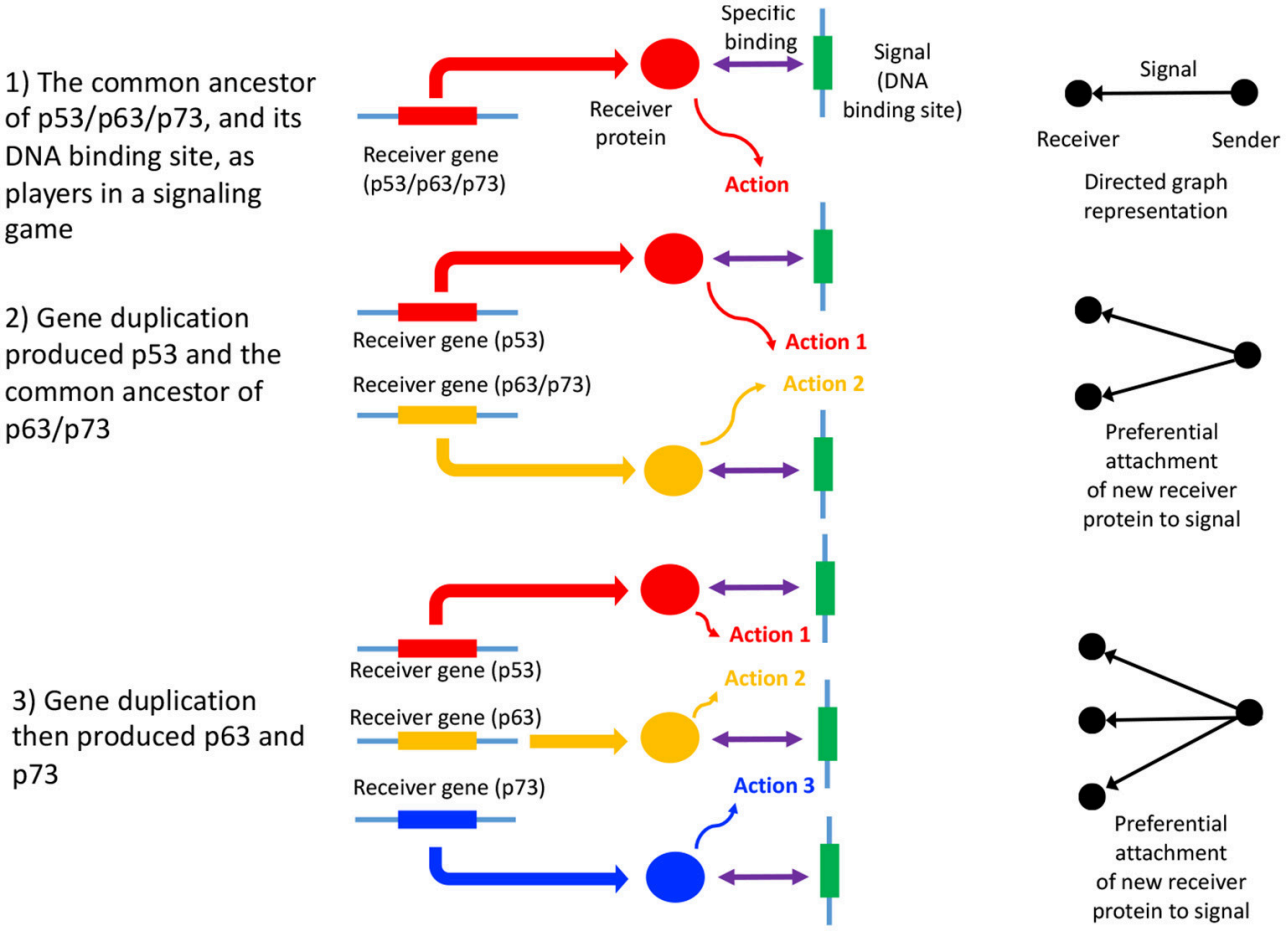
Gene duplication of p53, p63, and p73 as a signaling game, and GRN growth. An illustrative example of a signaling games view of network growth is provided by the paralogs p53, p63, and p73, which code for transcription factors, p53 being of critical importance in many cancers (Joerger and Fersht, 2006). Here, p53 and the common ancestor of p63/p73 duplicated (2), followed by the duplication and divergence of p63 and p73 (Lu et al., 2009; Belyi et al., 2010) (3). The signal is the DNA binding site, while the receivers are the p53, p63, and p73 proteins (here the sender is the protein coding gene downstream of the DNA binding site). The receiver protein undergoes an action upon binding to the DNA binding site (the signal), which consists of the recruitment of additional transcription factors, and contribution to the assembly of the transcription initiation complex (Nogales et al., 2017). The gene products of p53, p63, and p73 mostly bind to the same DNA binding sites (Smeenk et al., 2008), thus each signal (and ultimately sender gene) has acquired two new binding partners, in addition to the original interaction with the gene product of the common ancestor of p53/p63/p73. This is a form of preferential attachment, which should influence network topology as the number of genes increase by duplication, as illustrated to the right of the figure. The signaling games perspective allows us to better understand scenarios where there is a conflict of interest between the genome, and a selfish entity such as a selfish element, a cancer or a virus. When there is a conflict of interest, a deceptive signal is expected to be emitted by the sender (Crawford and Sobel, 1982) (the selfish entity). Here, the DNA binding site of the selfish entity will mimic that of canonical DNA binding sites associated with normal cellular function, “tricking” a transcription factor to bind to it, and altering the transcription of the sender gene (or alternatively abolishing transcription factor binding). Examples include *cis*-regulatory mutations in cancer (Poulos et al., 2015).

In summary, the process of gene duplication, tempered by signal and action genesis can be thought of as a driver of preferential attachment in shaping the topology of gene networks, in which information asymmetry between senders and receivers is expected to play an indelible role. Figure 1 illustrates a basic mechanism whereby signal genesis may lead to preferential attachment during the growth of a PPI network. Topological features expected to hint at this process include: (i) the degree distribution, (ii) hierarchicity, (iii) assortativity and many others; they require powerful statistical and algebraic tools—covered in the later sections, where it is assumed that genome evolution is a complex process involving diverse groups of mutations such as insertions, deletions, conversions, duplications, transpositions, translocations, and recombinations, and that it is further affected by selective constraints and effective population size and other factors such as the environment. With recent understanding of large scale cellular networks (regulatory, metabolic, protein-protein interactions) one must now aim at investigation between the evolutionary rates of a gene mutations and its effects on the network topology using mathematical models and analytics (see Wagner, 1994). For instance, combining sequence analysis in a single genome and its close relatives, one can infer the rate and tempo of the evolutionary dynamics acting on the genome, and the resulting effects on the network's algebraic structures. We provide an example of how evolution by duplication leads to a preferential attachment mode of gene network growth in Figure 2, using the duplication of the p53 gene, and its paralogs p63 and p73—all transcription factors regulating pathways involved in related phenotypes of somatic or developmental surveillance and interacting with similar family of genes (e.g., MDM2 or MDMX), as illustration^1^.

Note that these abstract models generate refutable hypotheses that need experimental verification and support from mechanistic explanations. However, unfortunately, the biochemical processes involved in the hypothesized preferential attachment dynamics are not fully understood. For example, the duplication processes are often driven by Non-Homologous End Joining (NHEJ), a pathway that repairs double-strand breaks in DNA. To guide repair, NHEJ typically uses short homologous DNA sequences called microhomologies, which are often present in single-stranded overhangs on the ends of double-strand breaks (Chang et al., 2017). When the overhangs are perfectly compatible, NHEJ usually repairs the break accurately. However, imprecise repair can lead to inappropriate NHEJ resulting in translocations, duplications, and rearrangements (Rodgers and McVey, 2016), which add to variational processes that are random but not memoryless. Perhaps some of such hypotheses may need to be carefully examined using cancer genome data such as The Cancer Genome Atlas (TCGA), and models of tumor progression. This analysis may also explain efficacy of certain therapeutic interventions in cancer as well as their failures via drug and immuno resistance.

## 2. Network Analysis

In this section, in order to address the potential impact of information asymmetry on network evolution, it is first necessary to discuss fundamentals of graphs (in particular directed and weighted graphs), a mathematical formalism used in the study of biomolecular networks, as well as other related important topics. Consider a set of entities, denoted *V* and a set of binary relations between the entities *E* ⊆*V* × *V*. When *V* denotes biomolecules and *E* denotes interactions between them (e.g., regulations, proximity, synteny, etc.), the resulting graph represents a biomolecular network. One important advantage of graphs is that they have an intuitive graphical representation. Such networks evolve over time with additions and deletions to the sets *V* and *E*. In order to create a bridge to algebraic approaches, we extend the standard combinatorial definition by endowing it with additional maps.

Formally, a graph is a pair of sets *G* = (*V, E*) where *V* are the vertices (nodes, points) and *E*⊆*V*×*V* are the edges (arcs), respectively. When *E* is a set of unordered pair of vertices the graph is said to be undirected or simple. In a directed graph (which could result from information asymmetry, for example) *G* = (*V, E, o, t*), *E* consists of an ordered set of vertex pairs, i.e., for each edge *e*∈*E*, *e* → (*o*(*e*), *t*(*e*)) where *o*(*e*) is called the origin of the edge *e* and *t*(*e*) is called the terminus of the edge e (Serre, 1980; Biggs, 1993). A graph is weighted if there is a map (weighting function, *w*:*E*→*R*_+_) assigning to each edge a positive real-valued weight. Weighting can represent the strength of a signal in a sender-receiver interaction, for example.

If *G* = (*V, E*, ·, ·) and *G*′ = (*V*′, *E*′, ·, ·) are two graphs such that *V*′⊆*V* and *E*′⊆*E*∩(*V*′ × *V*′), then *G*′⊆*G*, *G*′ is a *subgraph* of *G*. If *E*′ = *E*∩(*V*′ × *V*′) (*E*′ contains every edge in *e*∈*E* with *o*(*e*), *t*(*e*)∈*V*′) then *G*′ is an *induced subgraph* of *G*. *G*′ and *G* are *isomorphic* (*G*′≡*G*) if there is a bijection *f*:*V*′ → *V* with (*u, v*)∈*E*′⇔(*f*(*u*), *f*(*v*))∈*E*, ∀*u, v*∈*V*′.

### 2.1. Topological Properties

A network's properties are governed by its topology, such as the degree distribution, clustering coefficients, motifs, assortativity, etc. Comprehensive treatments for general networks can be found in Thulasiraman et al. (2015) and Loscalzo and Barabási (2016), and for more in-depth treatment regarding biomedical networks in Loscalzo et al. (2017). Here we discuss these properties in the context of biomolecuar networks, more specifically with respect to information asymmetry. The Supplementary Material contains a more complex combinatorial and algebraic graph theoretic approach.

#### Degree Distribution

The degree of a vertex *v*, deg(*v*), is the number of edges that connect the vertex with other vertices. In other words, the degree is the number of immediate neighbors of a vertex. In directed graphs the in-degree and out-degree of a vertex can be defined as the number of incoming and outgoing edges, respectively. Let *n*_*k*_ be the number of vertices of degree *k* and |*V*| = *N*, the total number of vertices in the graph and |*E*| = *M*, the total number of edges in the graph. Note that $\underset{k}{\sum }n_{k}=N$ and $\sum kn_{k}=\underset{v\in V}{\sum }\deg\left(v\right)=2|E|=2M$. The degree distribution is the fraction of vertices of degree *k*, *P*(*k*) = *n*_*k*_/*N*, and two isomorphic networks will have the same degree distributions (though not necessarily the converse). Thus, the degree distributions can tell a great deal about the structure of a family of networks. For example, if the degree distribution is singly peaked, following the Poisson (or its Gaussian approximation) distributions, the statistical properties of the nodes can be described by the average degree $\langle k\rangle =\underset{k}{\sum }kP\left(k\right)=2M/N$. The graph is said to be *sparse*, if 〈*k*〉 = *o*(log*N*) (or *M* = *o*(*N*log*N*)). Biomolecular networks are usually sparse, which can be fruitfully exploited in their algorithmic analysis. We can talk of *typical* nodes of the networks as being those that have degree distribution as those within 1 to 2 standard deviations from the average, while, with probability decreasing exponentially, it is possible to find nodes with a degree much different from the average. While power-law degree distributions follow a completely different pattern: they are *fat-tailed*; the majority of the nodes have only a few neighbors, while many nodes have a relatively large number of neighbors. The highly connected nodes are known as *hubs*.

#### Distance Metrics

One of the most fundamental metrics is the *distance* on a graph. First we define a *walk* of length *m* in a graph *G* from a vertex *u* to *v* as a finite alternating sequence of vertices and edges 〈*v*_0_, *e*_1_, *v*_1_, *e*_2_, …, *e*_*m*_, *v*_*m*_〉, such that *o*(*e*_*i*_) = *v*_*i* − 1_ and *t*(*e*_*i*_) = *v*_*i*_, for 0 < *i* ≤ *m*, such that *u* = *v*_0_ and *v* = *v*_*m*_. Then the number of edges traversed in the shortest walk joining *u* to *v* is called the *distance* in *G* between *u* and *v* denoted by *d*(*u, v*). If there is a walk from *u* to itself, then we say that the set of vertices (respectively edges) form a cycle. The smallest number of *m* edges in a walk from *u* to itself is called a cycle of length *m*. The girth *g*(*G*), is the shortest cycle in *G*. A walk whose vertices are distinct is called a (simple) *path*.

The concept of a walk allows us to define other properties of the graph. A graph *G* = (*V, E, o, e*) is said to be *connected*, if any two vertices are the extremities of at least one walk. The maximally connected subgraphs are called the *connected components* of *G*. A giant component is a connected component containing a significant fraction of the nodes. The maximum value of the distance function in a connected graph is called the *diameter* of the graph. Frequently real life networks have a small diameter and are said to exhibit the *small world phenomenon*. For many biomolecular networks the average distance between two nodes depends logarithmically on the number of vertices in the graph.

Additionally, a *complete graph* *G* is the undirected graph, in which each vertex is a neighbor of all other vertices; deg(*v*) = *N* − 1, ∀*v*∈*V*; or equivalently, each distinct pair of vertices are connected (or are adjacent) by a unique edge. *G* is then denoted as *K*_*N*_. A *clique* in an undirected graph is a subset of vertices such that its induced subgraph is complete. Additional combinatorial invariants of graphs useful in the analysis of networks can be defined (see Supplementary Material for details).

#### Expanding Constants

Let *G* = (*V, E*, ·, ·) be an undirected graph. Then for all *F*⊂*V*, the *boundary* ∂*F* is the set of edges connecting *F* to *V*\*F*. The *expanding constant*, or *isoperimetric constant* of *X* is defined as,

$$\begin{array}{l} h\left(X\right)=\underset{\emptyset \neq F\subset V}{\min}\frac{\left|\partial F\right|}{\min\left\{\left|F|,|V\backslash F\right|\right\}}. \end{array}$$

For a biomolecular network, then, the invariant *h*(*X*) measures the quality of the network with respect to the flow of information within it, (e.g., via chemical reactions, or signaling). A larger *h*(*X*) implies better expansion, faster mixing, faster partitioning, and many other related properties that may give the network a selective advantage.

Using various combinatorial algorithms devised for the study and analysis of biomolecular networks, one may compute *h*(*X*) to determine their complexity. However, a precise characterization of *h*(*X*) itself is an intractable (i.e., NP-complete) problem. Isoperimetric inequalities give bounds on *h*(*X*) in terms of a related algebraic invariant, γ(*X*) – called its *spectral gap*, determination of which has complexity *O*(|*V*|)^*c*^, where *c* is at most 3; furthermore, *c* = 1 for many sparse graphs. We give isoperimetric bounds and results applicable to biomolecular networks in the Supplementary Material, where we also introduce a local Cheeger constant. We also introduce algebraic invariants in section 2.2.

#### Clustering and Clustering Coefficients

**Figure 4:**
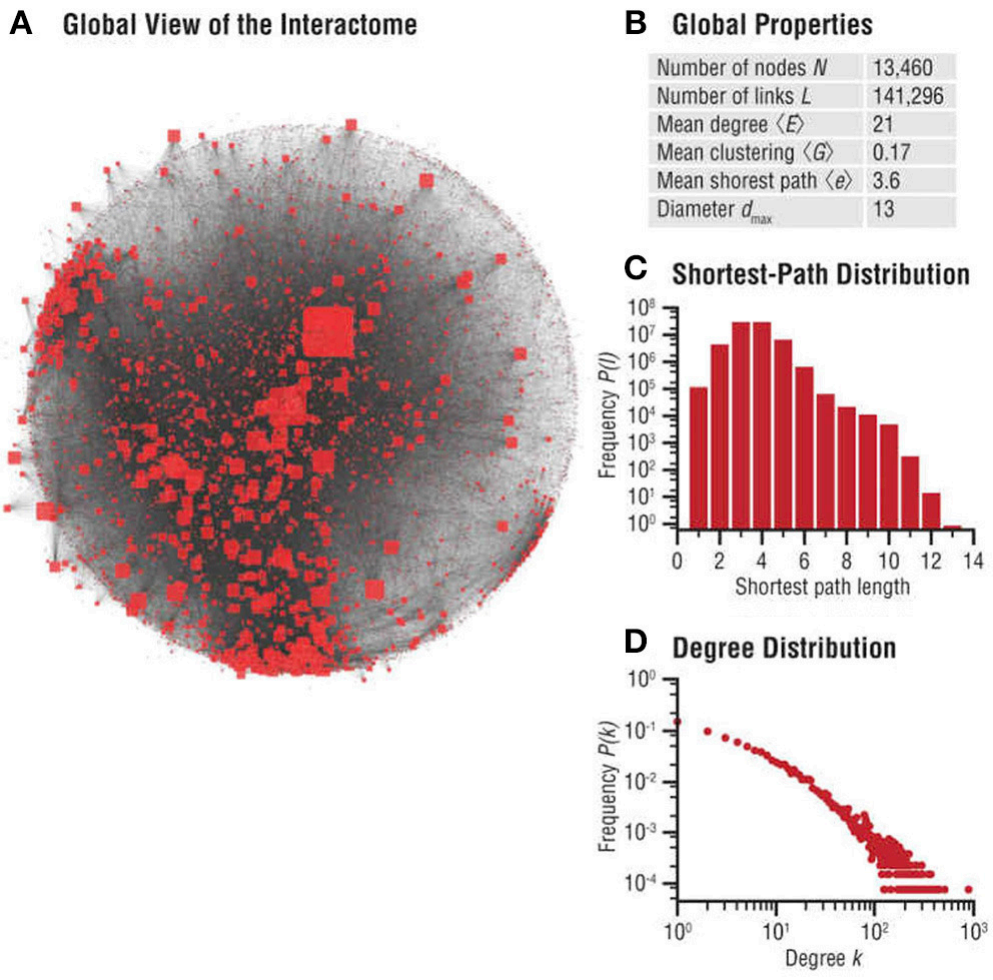
Interactome networks used in the study of diseases. Undesirable interactions within a biomolecular network result in various disease states. Disease neighborhoods within the interactome can then be mapped to understand the progression of the disease [details can be found in Loscalzo et al. (2017)]. The progression of cancer has been studied using analysis of functionalization of oncogenes and dysfunctionalization of tumor suppressor genes via copy number fluctuations, but much more can be learned from the topological features of these genes in their interaction neighborhood. This illustration is from Figure 2.3 **(A–D)** of Menche and Barabási (2017). **(A)** Global map of the interactome, illustrating its heterogeneity. Node sizes are proportional to their degree, that is, the number of links each node has to other nodes. **(B)** Basic characteristics of the interactome. **(C)** Distribution of the shortest paths within the interactome. The average shortest path is 〈*d*〉 = 3.6. **(D)** The degree distribution of the interactome is approximately scale-free (reproduced with permission from the publisher and authors of Menche and Barabási, 2017).

Biomolecular networks are modular, forming communities and hierarchies, likely to have been sculpted by EBD (Evolution by Duplication). To study these local structures in network science, one may perform *community* analysis, which aims to identify a group of nodes that have a higher probability of connecting to each other than to nodes from other communities [see for example (Pellegrini, 2019)]. These can be explained by our game theoretic formalism, and local Nash equilibria (see Massey and Mishra, 2018). Various notions such as *k*-cliques, *k*-clubs, and *k*-clans have been developed to detect communities, but they are ultimately closely connected to the problem of finding cliques and consequently, do not generally lend themselves to any reasonable algorithm other than brute-force enumeration. However, even detecting communities approximately may prove valuable for general evolutionary studies, since these biomolecular network communities determine how specific biological functions are encoded in cellular networks—and are thus subjected to Darwinian selective pressure, since these players are likely to have formed communities in the first place to carry out specific cellular functions (see Hartwell et al., 1999), maximizing the utility of the cell. Figure 4 highlights significant evidence that communities play an important role in human disease networks (see Loscalzo et al., 2017).

Usually a simpler approach is commonly employed and deals with the problem of *clustering* in a graph, which seeks to partition the graph into disjoint subgraphs such that nodes in each such subgraph are “closer” to the other nodes in the same subgraph, while they are “farther” from the nodes of other subgraphs. Hierarchical clustering algorithms have been developed to uncover communities (approximately) in polynomial time and depend upon the *similarity matrix* (*x*_*ij*_), where the entry *x*_*ij*_ equals the distance between node *i* and node *j*. Among the classical algorithms are included those by Girvan and Newman (2002). Other related algorithms include those for random-walk betweenness and network centrality.

The *local clustering coefficient* captures the degree to which the neighbors of a given node link to each other. In general, for undirected graphs, the *local clustering coefficient* *C*_*i*_ of node *i* with degree *k*_*i*_ is defined as

$$\begin{array}{l} C_{i}:=\frac{L_{i}}{k_{i}\left(k_{i}-1\right)/2} \end{array}$$

where the numerator *L*_*i*_ is the actual number of connections between *k*_*i*_ immediate neighbors of *i*, and the denominator is the number of connections if the neighbors formed a complete graph (i.e. a clique). Note that an undirected complete graph *K*_*k*_*i*__ of *k*_*i*_ nodes has *k*_*i*_(*k*_*i*_ − 1)/2 edges. Thus, a fully clustered node will have *C*_*i*_ = 1 and for completely isolated node *C*_*i*_ = 0. We can define the *(average) clustering coefficient* of the whole network with *N* nodes as

$$\begin{array}{l} \langle C\rangle =\frac{1}{N}\sum C_{i}. \end{array}$$

The clustering coefficients can be used to characterize a network's *modularity*, as discussed later (in section 3) in detail. For weighted graphs and directed graphs (as in the context of information asymmetry), a similar formalism is discussed in the Supplementary Material.

#### Subgraphs and Motifs

Biomolecular networks have been found to contain network *motifs*, representing elementary interaction patterns between small subgraphs that occur substantially more often than as predicted by a completely random network of similar size and connectivity. The presence of such motifs is usually explained by an evolutionary process that can quickly create (usually by a variation involving duplication) or eliminate (usually by a selection process that favors pseudogenization and complementation) regulatory interactions in a fast evolutionary time scale—relative to the rate at which individual genes mutate. It is usually hypothesized that the underlying evolutionary processes are convergent. Thus efficient algorithms to detect such motifs are important in the analysis of biomolecular networks. These algorithms focus on estimating how much more frequently a subgraph isomorphic to a motif graph (with *n* vertices and *m* edges) occurs relative to what would be expected by pure chance.

The number *N*_*mn*_ of subgraphs with *n* nodes and *m* interactions expected of a network of *N* nodes can be estimated from the two key topological parameters of a complex network—namely the power-law exponent β and the hierarchical exponent α as we discuss in Equations (1 and 2) below. In general the subgraph motifs can be classified in two types: Type I motifs are those where (*m* − *n* + 1) α − (*n* − β) < 0, and type II subgraph motifs are those that satisfy the reverse inequality. One can determine their numbers *N*^*I*^ and *N*^*II*^ approximately as a function of (*m* − *n* + 1) α − (*n* − β) and *n*_*max*_, the degree of the most connected node in the network. One can show that *N*^*I*^>>*N*^*II*^. One can also show that the relative number of Type II subgraphs is vanishingly small compared to Type I.

### 2.2. Algebraic Invariants and Spectrum

The intuitive pictorial/combinatorial representation of graphs is an extremely useful aid to their understanding. However, computing the topological properties of graphs combinatorially is computationally challenging especially when the size of the graph becomes large. As noted earlier, indeed, most combinatorial algorithms on biomolecular networks such as on PPI networks and GRNs are computationally complex problems (most of them fall in the NP-complete complexity class) (Karp, 2011). Therefore, in order to carry out any quantitative and computational analysis, graphs are better represented as algebraic objects. This representation allows us to use linear algebra and mathematical analysis techniques. The key to this representation is the adjacency matrix *A*(*G*). It is defined as {0, 1}^*n*×*n*^ matrix in which, *A*_*ij*_ = 1 if the vertices *i* and *j* are connected [∃*e*∈*E, o*(*e*) = *i, t*(*e*) = *j*] and 0 otherwise. The matrix is symmetric if the graph is undirected. For weighted graphs we can assign weights *w*_*ij*_ for existing edges. Networks that incorporate information asymmetry are directed, and the analysis becomes more complex. We refer to the Supplementary Material for this treatment.

Algebraic properties provide us with tools to deduce various properties of the biomolecular networks. In particular, the spectral representation of the graph is of importance for a number of applications such as graph classification, diffusion, expansion and mixing (see the Supplementary Material). We can think of the adjacency matrix *A* as operating on the space *V* = *C*^*n*^ of complex *n*-tuples written as column vectors *x*,*y* as follows *Ax*→*y*. It can be shown that there are directions left invariant in this space. That is to say, *Ax*_*i*_ = λ_*i*_*x*_*i*_ where λ_*i*_ are the eigenvalues and corresponding *x*_*i*_ the eigenvectors (spanning invariant directions) of the adjacency matrix for 1 ≤ *i* ≤ *n*. The spectrum of the graph *G* is defined as the collection of eigenvalues of the adjacency matrix Spec(*G*) = Spec(*A*) = λ_1_, .., λ_*n*_. Naturally, if *A* is a real symmetric matrix, then the eigenvalues of *A* are real.

In particular, one algebraic invariant of the graph is the *spectral gap* γ(*G*). It can be shown that the spectral gap gives excellent bounds on a combinatorial invariant, the Cheeger constant *h*(*G*). Since information asymmetry leads to directed, weighted graphs, some of which are bipartite networks, we discuss these deeper algebraic analytics in the Supplementary Material.

## 3. Network Evolution

Starting with the seminal work of Erdös and Rényi (1959), a number of mathematical frameworks have been developed to model the “evolution” of graphs, covering the family of biomolecular networks. These frameworks may prove useful in explaining why most biological networks have certain non-obvious properties: namely, (i) The small world property; (ii) High clustering coefficients (varying with degree distribution); (iii) Emergence of “hubs.” Such network models are ultimately expected to capture various observed properties of biomolecular networks, and the evolutionary trajectories leading up to them. The novel factor of information asymmetry, modeling genes as players, may also be incorporated, using the basic principles outlined in the Introduction, and Figures 1, 2.

### 3.1. Random Network Models

#### Erdös and Rényi Model

The Erdös and Rényi model of random graphs [ER-graphs, denoted *G*(*n, p*)] is characterized by two parameters, the number of vertices in the network *N* and the fixed probability of choosing edges *p* (Erdös and Rényi, 1959). The graph *G* is generated by choosing *N* vertices and connecting each pair of vertices with probability *p*. The model yields a network with approximately pN2=O(pN2) randomly distributed edges. The probability of choosing a specified graph *G* with *N* vertices and *e* edges is therefore Mepe(1-p)M-e, where M=N2= the maximum number of possible edges connecting *N* vertices.

It can be shown that in such random graphs the average vertex degree is 〈*k*〉 = *p*(*N* − 1) = *O*(*pN*). The diameter of such a graph is *d* = ln *N*/ln 〈*k*〉 ≈ ln *N*/(ln *N* − *ln*(1/*p*)) which is small compared to the graph size. Thus, random graphs exhibit “the small world property.” The degree distribution for ER graphs is a binomial distribution P[deg(u)=k]=(N-1)kpk(1-p)N-k-1, which for large *N* (relative to 1/*p*: where *N* = λ/*p*) converges to the Poisson distribution $P\left[\deg\left(u\right)=k\right]=e^{-\lambda }\frac{\lambda^{k}}{k!}$. Then the local clustering coefficient is *C*_*i*_ = *p* is independent of the degree of the node and the average clustering coefficient *C* = *p*/*N* scales with the network size. Therefore, the standard ER random model seems not to capture either the properties of degree distribution or the clustering coefficient of biomolecular networks.

Typically, an ER random graph model is used as a “null model” for the evolutionary process. However, while deviations from randomness are frequently used as evidence for the direct action of natural selection, often non-randomness may reflect neutrally generated (non-adaptive) emergent phenomena (Massey, 2015). We emphasize here that many topological features of biomolecular networks are unlikely to be directly selected for, but instead are a side-product of network growth, and decay, captured by the dynamics of edge and node addition and removal.

#### Small World Model

Biomolecular networks have features that are not captured by the Erdös and Rényi random graph model. As we have seen, random graphs have a low clustering coefficient and they do not account for the formation of hubs. To rectify some of these shortcomings, the *small world model* or popularly known as the *six degree of separation model* was introduced as the next level of complexity for a probabilistic model with features that are closer to real world networks (Watts and Strogatz, 1998; Watts, 1999). The evolution and dynamics of such networks have been discussed in detail (Watts, 2003), in particular in the diseases propagation literature (Dodds and Watts, 2005).

In this model, the graph *G* of *N* nodes is constructed as a ring lattice, in which, (i) first, *wire*: that is, connect every node to *K*/2 neighbors on each side and (ii) second, *rewire*: that is, for every edge connecting a particular node, with probability *p* reconnect it to a randomly selected node.

The average number of such edges is *pNK*/2. The first step of the algorithm produces local clustering, while the second dramatically reduces the distance in the network. Unlike random graphs, the clustering coefficient of this network *C* = 3(*K* − 2)/4(*K* − 1) is independent of the system size. Thus, the small world network model displays the small world property and the clustering of real networks, however, it does not capture the emergence of hubby nodes (e.g., p53 in biomolecular networks)(part of one of the eight open problems that we formulate in section 4).

### 3.2. Scale-Free Network Models

Most biomolecular networks are hypothesized to have a degree distribution, described as *scale-free*. In a scale free network the number of nodes *n*_*k*_ of degree *k* is proportional to a power of the degree, namely, the degree distribution of the nodes follows a *power-law*

(1) $$\begin{array}{r} n_{k}=k^{-\beta }, \end{array}$$

where β>1 is a coefficient characteristic of the network (Barabási and Albert, 1999). Unlike in random networks, where the degree of all nodes is centered around a single value – with the probability of finding nodes with much larger (or smaller) degree decaying exponentially, in scale-free networks there are nodes of large degree with relatively higher probability (*fat tail*). In other words, since the power low distribution decreases much more slowly than exponential, for large *k* (heavy or fat tails), scale-free networks support nodes with extremely high number of connections called “hubs.” Power law distribution has been observed in many large networks, such as the Internet, phone-call maps, collaboration networks, etc. (Képès, 2007; Barabási, 2009; Loscalzo and Barabási, 2016). A caveat to these reports is that inappropriate statistical techniques have often been used to infer power law distributions, and alternative heavy tailed distributions may fit the data better (Clauset et al., 2009). However, the power law is a useful approximation that allows mechanisms of network growth to be explored, such as preferential attachment, discussed next, while the examination of alternative heavy tailed distributions is set as an Open Problem.

#### Preferential Attachment

The original model of *preferential attachment* was proposed by Barabási and Albert (1999). The scheme consists of a local *growth rule* that leads to a global consequence, namely a power law distribution. The network grows through the addition of new nodes linking to nodes already present in the system. There is higher probability to preferentially link to a node with a large number of connections. Thus, this rule gives more preferences to those vertices that have larger degrees. For this reason it is often referred to as the “rich-get-richer” or “Matthew” effect. This can be formulated as a game theoretic problem originating from information asymmetry and associated Nash equilibrium, discussed in the Open Problems.

With an initial graph *G*_0_ and a fixed probability parameter *p*, the preferential attachment random graph model *G*(*p, G*_0_) can be described as follows: at each step the graph *G*_*t*_ is formed by modifying the earlier graph *G*_*t* − 1_ in two steps – with probability *p* take a *vertex-step*; otherwise, take an *edge-step*:

- *Vertex step:* Add a new vertex *v* and an edge {*u, v*} from *v* to *u* by randomly and independently choosing *u* proportional its degree;
- *Edge step:* Add a new edge {*r, s*} by independently choosing vertices *r* and *s* with probability proportional to their degrees.

That is, at each step, we add a vertex with probability *p*, while for sure, we add an additional edge. If we denote by *n*_*t*_ and *e*_*t*_ the number of vertices and edges respectively at step *t*, then *e*_*t*_ = *t* + 1 and $n_{t}=1+\overset{t}{\underset{i=1}{\sum }}z_{i}$, where *z*_*i*_'s are Bernoulli random variables with probability of success = *p*. Hence the expected value of nodes is 〈*n*_*t*_〉 = 1 + *pt*.

It can be shown that exponentially (as *t* asymptotically approaches infinity) this process leads to a scale-free network. The degree distribution of *G*(*p*) satisfies a power law with the parameter for exponent being $\beta =2+\frac{p}{2-p}$. Scale-free networks also exhibit *hierarchicity*. The local clustering coefficient is proportional to a power of the node degree

(2) $$\begin{array}{r} C\left(k\right)\approx k^{-\alpha } \end{array}$$

where α is called the *hierarchy coefficient*.

This distribution implies that the low-degree nodes belong to very dense sub-graphs and those sub-graphs are connected to each other through hubs. In other words, it means that the level of clustering is much larger than that in random networks.

Consequently, many of the network properties in a scale-free network are determined by local structures—namely, by a relatively small number of highly connected nodes (hubs). A consequence of this structure of the scale-free network is its extreme robustness to failure, a property also displayed by biomolecular networks and their modular structures. Such networks are highly tolerant of random failures (perturbations); however, they remain extremely sensitive to targeted attacks.

#### Assortativity Network Model

*Assortative mixing* refers to the property exhibited by a preference of nodes to attach to similar (respectively, dissimilar) nodes; for example, high-degree vertices exhibit preference to attach to high-degree (resp. low-degree) vertices. Network models, discussed earlier and including the preferential attachment model, do not capture such important properties exhibited by real biomolecular networks (Girvan and Newman, 2002). Assortativity can be measured by the Pearson correlation coefficient *r* of degrees of linked nodes (Girvan and Newman, 2002). A positive correlation means connections between nodes of similar degree (assortativity) and a negative correlation means connections between nodes with different degree (disassortativity). Unlike technological networks and social networks (that show assortative mixing), biological networks appear to evolve in a disassortative manner.

GRNs are represented by directed graphs, and all biomolecular networks may be represented as directed graphs when the factor of information asymmetry is introduced (Figures 1, 2). Assortative mixing can be generalized to directed biological graphs (Piraveenan et al., 2012). For directed networks two new measures, in-assortativity and the out-assortativity , can be defined measuring the correlation between the in-degree *r*_*in*_ and out-degree *r*_*out*_ of the nodes respectively. Biological networks, which have been previously classified as disassortative, have been shown to be assortative with respect to these new measures. Also it has been shown that in directed biological networks, out-degree mixing patterns contain the highest amount of Shannon information, suggesting that nodes with high local out-assortativity (regulators) dominate the connectivity of the network (Piraveenan et al., 2012). The occurrence of assortativity in social networks has been attributed to a process of homophily [that is people tend to associate with others on the basis of ethnicity, religion, sports preferences etc. (McPherson et al., 2001; Newman, 2003a)]. The mechanisms that give rise to assortativity in biomolecular networks likely arises by a similar proximate mechanism of like nodes forming edges with like nodes, but the ultimate cause(s) remain unclear.

#### Duplication Model

Our earlier discussions suggest that biomolecular networks exhibit power-law degree distribution. However, unlike other complex networks, such as the Internet, the growth exponent of biomolecular networks typically falls into a lower range 1 < β < 2, as opposed to β≥2. This difference has been suggested to have resulted from evolution by gene duplication dominating the evolutionary mechanism (Chung et al., 2003). We have already discussed the duplication phenomenon based on information asymmetry in GRNs in section 1. Various biomolecular networks have been studied using a partial duplication process, which proceeds in the following manner: Let the initial graph *G*_0_ have *N*_0_ vertices. In each step, *G*_*t*_ is constructed from its previous graph *G*_*t* − 1_ as follows: A random vertex *u* is selected. Then a new vertex *v* is added in such a way that for each neighbor *w* of *u*, a new edge (*u, w*) is added with probability *p*. The process is then applied repeatedly. The full duplication model is simply the partial model with *p* = 1.

It has been shown that as the number *N* of vertices becomes infinitely large (as is the case for most biomolecular networks), the partial duplication model with selection probability *p* generates power-law graphs with the exponent satisfying the transcendental equation (Chung et al., 2003)

$$\begin{array}{l} p\left(\beta -1\right)=1-p^{\beta -1}, \end{array}$$

whose solution determines the scale-free exponent β as a function of *p*. In particular, if 1/2 < *p* < 1 then β < 2.

For illustrative purposes, we describe below an abstract gene network growth model incorporating the processes of gene duplication and deletion, as described above ( Mishra and Zhou, 2004; Zhou, 2005). Using a Markov chain model the following features were investigated: (i) the origination of the segmental duplication; (ii) the effect of the duplication on the genome structure; and (iii) the role of duplication and deletion process in the genomic evolutionary distance. Unlike standard models of stationary Markov chain models, most processes in evolutionary biology belong to the group of non-stationary Markov processes, in which the transition matrix changes over time, or depends upon the current state.

This model results in the neutral emergence of scale-free degree distributions. It shows that the genomes of different organisms exhibit different network properties, likely reflecting differences in the rates of gene duplication and deletion (Mishra and Zhou, 2004). The additional factor of information asymmetry is likely to affect the nature of gene duplication in terms of gene identity and rate of duplication, and may provide additional explanatory power for differences in network properties. This analysis provides an example of how network topology can be used to provide insight into fundamental molecular evolutionary (neutral/Markov) processes in different species. Note that the model is relatively idealized, as it does not account for higher order interactions in a population involving: effective population size and allelic fixations; sex, diploidy, and sex-chromosomes (e.g., X and Y in mammals or W and Z in birds, etc.); surveillance and repair in somatic cells; embryonic lethality; homologous recombination, etc. The mathematical model explored here is kept simple to motivate the machinery from graph theory developed later.

#### Hierarchical Network Models

Another interesting model, introduced by Ravasz and Barabasi and dubbed the *hierarchical network model*, simulates the characteristics of many real life complex models and may be relevant. The resulting networks have modularity, a high degree of clustering, and the scale-free property. Modularity refers to the network phenomenon where many sparsely inter-connected dense subgraphs can be identified—“one can easily identify groups of nodes that are highly interconnected with each other, but have only a few or no links to nodes outside of the group to which they belong” (from Ravasz and Barabási, 2003).

A generative process for a hierarchical network model may be described as follows: For instance, consider an initial network *H*_0_ of *c* fully interconnected nodes (e.g., *c* = 5). As a next step, create (*c* − 1) replicas of this cluster *H*_0_ and connect the peripheral nodes of each replica to the central node of the original cluster to create *H*_1_ with *c*^2^ (e.g., *c*^2^ = 25) nodes. This step can be repeated recursively and indefinitely, thereby for any *k* steps the number of nodes generating the graph *H*_*k*_ with *c*^*k* + 1^ nodes. If the central nodes of *H*_0_ is called a *hub* and other nodes *peripheral*, then each recursion replicates additional copies of hubs and peripheral nodes.

One can carry out a recursive analysis and show that one obtains a power-law (i.e., scale-free) network with exponent $\beta =1+\frac{\ln\left(c\right)}{\ln\left(c-1\right)}.$ The local clustering coefficients (for the hub-nodes) follow $C\left(k\right)\approx \frac{2}{k}$. Also, one can show that this duplication feature of the evolutionary process leads to hierarchical behavior of the network. The resulting networks are expected to be fundamentally modular, in other words, the network can be seamlessly partitioned into collections of modules where each module performs an identifiable task, separate from the function(s) of other modules. One can also show that the average clustering coefficient on *N* nodes at any given stage is about *C* = 0.7419282.. (for *c* = 4), *C* = 0.741840 (for *c* = 5), and a constant for a fixed *c*, independent of *N* (see Ravasz and Barabási, 2003, and for exact computations Noh, 2003).

## 4. Open Problems and Future Challenges

The study of biomolecular networks is still a relatively young field and has thus far focused on a mechanistic perspective. As we begin to explore biomolecular networks from a more involved evolutionary view point, we encounter a large array of promising areas of investigation—most of which focus on how information asymmetries among the gene players ultimately sculpt the information flow, as necessary for an organism to navigate in a complex and fluctuating environment. Molecular evolution has classically been concerned with the dualism of selection and neutrality, however here we have highlighted a third important component, information asymmetry, and suggest a series of Open Problems that may help to begin to better understand its impact. The traditional approaches of phylogenetic study may be applied here, but examining specifically the family of species-specific biomolecular networks. Thus, mathematically we would need the networks to be aligned, motifs to be mapped to each other and network-distances to be correlated to deep evolutionary time. In order to account for the evolution by duplications, *orthologs* and *paralogs* of a gene (or gene families) are to be identified and connected to their roles in biochemical pathways. Ultimately, this analysis could be targeted at extracting the origin of various information-asymmetric signaling games and how they are stabilized in their Nash equilibria.

Key questions include whether signaling game characteristics differ between species. For example, species may differ in their average sender/receiver ratio, and the average complexity of signals produced (which may be indicated by protein size, variability in expression, and degree of post-translational regulation). Such differences may be linked to organismal complexity, variability in the environment and multicellularity. In so doing an overarching picture of how information is gathered from the environment, and how it is shared and distributed amongst gene players might be intimated. In particular, at its core this program requires an explanation of how features of genome evolution and structure might be algorithmically inferred from a network science perspective, as follows.

### 4.1. Algorithmic Complexity Issues

A key problem central to this program would be in detecting isomorphism mappings among pairs of graphs or subgraphs, a problem of infeasible algorithmic complexity (assuming *P*≠*NP*). We start with a discussion of these issues and cite heuristics that can tame the problem, albeit computing the solutions approximately.

#### Intractability: NP-Completeness

Many combinatorial optimization problems seem impossible to solve except by brute-force searches evaluating all possible configurations in the search space. They belong to a complexity class called NP-complete and include such problems as whether a graph has a clique of size *k*. Since finding certain recurrent motifs in a class of networks shares many computational characteristics of the clique problem and since it could be central to discovering important evolutionary signatures (e.g., EBD), it seems unlikely that it would be possible to characterize the evolutionary trajectories precisely—especially when the number of genes involved are in the thousands. See the Supplementary Material for additional discussions on graph representations and to derive their algebraic invariants, that provide bounds on complexity of algorithms possibly leading to excellent approximate results in the study of sparse complex networks (see Chung, 1997; Chung and Lu, 2006).

**Problem 4.A** *Classify various computational problems involved in detecting evolutionary trajectories of biomolecular networks and characterize their algorithmic complexity*.

**Problem 4.B** *Explore PTAS (Polynomial Time Approximation Schemes) for these problems—Especially when the graphs satisfy certain sparsity, modularity and/or hierarchy properties*.

#### Algebraic Approximation

As described earlier, many interesting topological features of a graph can be computed efficiently (on both sequential and parallel computers) from their descriptions in terms of adjacency matrices. The resulting spectral methods have found recent applications in complex networks (e.g., communication, social, Internet) (see Spielman, 1996, 2018; Chung, 1997, 2010; MacKay, 2003; Spielman and Teng, 2004, 2011, 2013, 2014; Chung and Lu, 2006). These methods are efficient (linear time complexity) for sparse graphs, whose number of edges is roughly of the same order as the number of vertices. Thus, they are well suited to biomolecular networks (for example for clustering, community detection, hubs, robustness, assortative mixing, spreading and mixing, closeness, isomorphism, among others).

Thus, spectral graph theory may be expected to have many applications in the analysis of biomolecular networks, most prominently, in clustering, graph similarity, and graph approximation, but also in smoothing analysis and sparsification. One can envisage that many, if not most, classical network algorithms in biomolecular networks can be made faster by spectral methods. Indeed, since most biomolecular networks are sparse—both in terms of sparse connections, and in precise algebraic sense (see the Supplementary Material), these algorithms likely lead to linear time algorithms. The smoothing analysis methods, as well as sparsification approximations are worth exploring in these contexts.

Another fruitful direction is in parallelizing these algorithms. As an illustration, in several studies of biomolecular networks it would be useful to identify when two networks *X*_1_ and *X*_2_ are “close.” We may wish to say that two networks are close if *Spec*(*X*_1_) and *Spec*(*X*_2_) are close—a computational problem that is polynomially computable (and efficiently parallelizable) (see Spielman and Teng, 2013). We can now give a mathematical formulation of this closeness, which can also be incorporated into phylogenetic studies. These biomolecular networks may be annotated with weights that are linear or quadratic approximation of relations, as common in these studies. These analyses may identify sub-networks that have been influenced by EBD, in concert with selection.

**Problem 4.C** *Classify various algebraic problems involved in detecting evolutionary trajectories of biomolecular networks and characterize their ability to approximate. Explore their practical implementations on sequential and parallel computers*.

### 4.2. Design Principles via Motif Analysis

The study of Systems Biology postulates that there are important design principles of biological circuits that provide a great deal of insight. The connections of gene and protein interaction networks are assumed to provide the necessary robustness and control to achieve cellular function in the face of chemical noise. However, it remains unclear how random variations alone provide such robustness. A possible explanation may come from a game-theoretic model that leads to stable equilibria and is expected to have precipitated from duplication of genes, interactions, and motifs. In addition, in principle, the dynamics of biomolecular sender-receiver signaling games should be reflected in network topologies, and so give rise to particular motifs. While the specific types of motifs expected to be observed remains to be developed further, some general principles can be identified. As discussed in section 1, the dynamics of signal genesis are driven by gene duplication, which affects overall network topology, in terms of the degree distribution. However, subgraphs consist of groups of senders and receivers, which likely have a related role in the cell, this may be tested by approaches outlined by Dotan-Cohen et al. (2009). The topology of these subgraphs contain localized motifs, which again reflect the addition and deletion of sender and receiver genes. The impact of information asymmetry is expected to lie in the Nash equilibria and associated utilities of sender-receiver interactions, which should be an influence on whether a new biomolecular interaction is established, or not.

#### Machine Learning

The biomolecular networks of interest are derived from highly noisy data e.g., CHIP-Chip, CHIP-Seq (for GRN), or co-localization or two-hybrid (for PPI) and consequently, the inferred edges of the network may miss certain genuine interactions or include several spurious interactions. Various machine learning algorithms (with false discovery rates, control, and regularization techniques) have been devised in order to improve the accuracy of such models. Biomolecular networks from related species (with ortholog and paralog analysis) are often combined to improve the accuracies and cross-validate results. The accuracies may be further ascertained via various local properties.

One important local property of networks is determined by so-called network motifs, which are defined as recurrent and statistically significant sub-graphs or patterns. Thus, network motifs are sub-graphs that repeat themselves in a specific network or even among various networks. Each of these sub-graphs, defined by a particular pattern of interactions between vertices, may reflect a framework in which particular functions are achieved efficiently. Indeed, motifs are of notable importance largely because they may reflect functional properties. They have recently gathered much attention as a useful concept to uncover structural design principles of complex networks. Although network motifs may provide a deep insight into the network's functional abilities, their detection is computationally challenging. Thus an important challenge for both experimental and computational scientists would be to study the evolutionary dynamics starting with the experimental data *ab initio*, as well as in improving the accuracy and efficiency of both the experimental and algorithmic techniques simultaneously.

**Problem 4.D** *Classify the species distributions of the different forms of heavy tailed distributions (e.g., power law, exponential, power law with exponential decay, lognormal), in different types of biomolecular network, and infer the mechanistic causes during network growth, and ultimate molecular evolutionary origins*.

**Problem 4.E** *Characterize the motifs in the biomolecular networks of closely related species starting with the noisy experimental data. Explain the structure of the motifs via their effect on the information flow. For instance, one may focus on DOR (Dense Overlapping Regulons) motifs and how they might have evolved from a simpler ancestral regulon (Alon*, *2006**)*.

**Problem 4.F** *Study Subgraph Isomorphism Algorithms (and heuristics) for sparse graphs and identify special cases most suitable for studying evolutionary trajectories, while relating them to biomolecular design principles*.

#### Network Alignment

Critical to the evolutionary studies, described above, is the topic of network alignment and subsequent network tree building, which may be used for the comparative approach, between species-specific networks. Networks may be aligned in a pairwise fashion to calculate similarity, and from this a distance matrix is calculated, and used for the construction of a network tree, showing the relationships between multiple networks. For example, in the case of meta-metabolic networks, such studies will reveal relationships between the meta-metabolic networks of different microhabitats. A plausible prediction is that the network tree should show convergent evolution in microbial communities from microhabitats with similar conditions (e.g., anaerobic habitats). Thus this approach could lead to a tool to study convergent evolution of microbial community structure in similar habitats (Goldford et al., 2018). The signaling games perspective promises a more complete view of the cooperation, and conflict, that is present in all microbial communities, and is expected to be reflected in the structure of meta-metabolic networks. In particular, cooperation will be indicated by honest signals, whereas conflict by the occurrence of deceptive signals, which are expected to include molecular mimics.

From an algorithmic point of view, one may employ any of the three types of network alignment approaches:

1. where node identity is known;
2. where node similarity can be determined (based on sequence similarity for example); and
3. where node identity is unknown, here only network topology is used for alignment.

**Figure 3:**
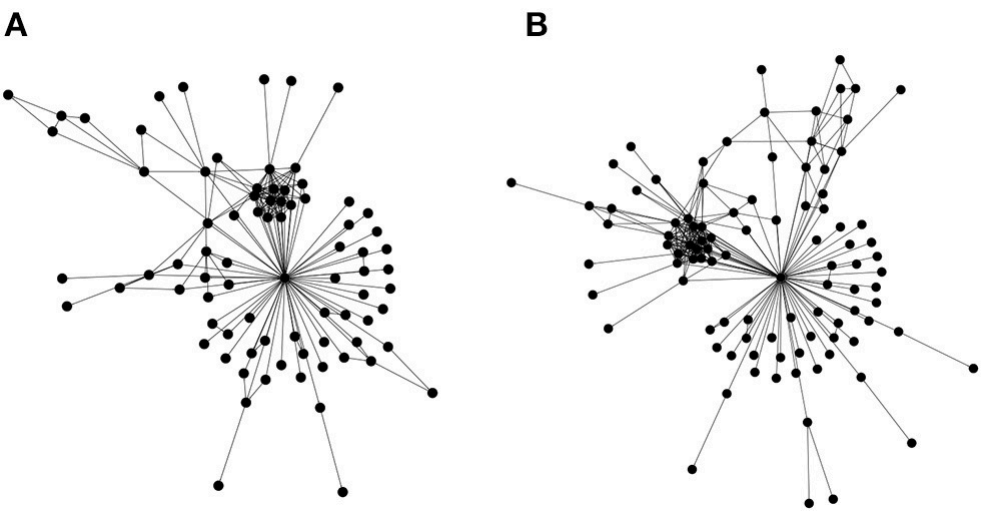
Topological alignment of networks. Similar biomolecular networks could be topologically aligned and compared in order to express an evolutionary distance, which may then augment the traditional approaches of phylogenetic study. In order to account for the evolution by gene duplications, genes (or gene families) are to be identified and connected to their roles in biochemical pathways. Such an approach would lead to a program to understand the critical role of information asymmetries in driving evolution. Network alignment, a core problem in this program, is computationally intractable. To sharpen our intuition, we illustrate the problem using the social networks of the Gospels of Matthew and Luke. These networks represent social interactions between characters in the gospels of Matthew **(A)** and Luke **(B)**. These were chosen as a basic test for topological alignment procedures, given that they share a similar number of nodes, and the highly connected node of Jesus. A straightforward test for the efficacy of a topological alignment algorithm therefore constitutes aligning both networks and verifying that the Jesus node from both networks is matched. Edge lists for the two social networks may be found in the Supplementary Materials.

The first is a straightforward edge alignment. However, a refined expression is required that incorporates similarities in edge widths in addition to the basic edge alignment (presence/absence of common edges between networks). Most effort in bioinformatics has gone into the second type network alignment, where there is partial information regarding node identity (for example Kalaev et al., 2005; Pinter et al., 2005). There do exist some first generation heuristics that utilize the third type of alignment approach (only topology) (Kuchaiev and Przulj, 2011), but the underlying graph isomorphism problem is known to be #P-complete. But these heuristics, as would be expected, do not work well—a straightforward test for this problem is applying them to align the social networks of the Gospels of Luke and Matthew (Figure 3)—the Jesus node should always align, as it is rather obvious topologically; but often leads to failure.

**Problem 4.G** *Classify and characterize the graph alignment algorithms*.

### 4.3. Somatic Evolution and Cancer

Network analysis is used in disease studies, but there have been more focused studies with applications to disease processes in cancer. In Figure 4 we show part of an interactome network useful in deciphering aberrant interactions in diseases (Figure 2.3 from Loscalzo et al., 2017). Cancer is a complex disease, but governed by somatic genomic evolution, as propelled by mutation. Thus as a consequence, GRNs may be used to better understand cancer susceptibility, map its progression, design better tailored therapies, and better understand the evolution of endogenous anti-cancer strategies. Cancer genes are often network hubs (Karimzadeh et al., 2018), as they are often involved in critical developmental pathways. But a better network analysis will shed light on many natural questions: Why is it so? How does this come about from the process of network growth over evolutionary time? What clues do they provide to understand the somatic evolution in cancer and its progression?

During cancer progression, the disease reduces a cell's healthy genome into an aberrant mutant, where cancer eventually leads to metastasis, ultimately resulting in death of the patient. The healthy cells in the patient may be thought to possess a normal network, that is a gene network that engenders health and well-being. Cancer progression is reflected by a dynamic change of the normal network into an aberrant network. The aberrant network manifests itself by tumorigenesis, and finally metastasis. There is a substantial literature enumerating the identity of oncogenes and tumor suppressor genes, which aberrantly gain function (e.g., amplification of copy number) or lose function (e.g., deletion in copy number, hemi- or homo-zygously), respectively. They modify the cell biology of cancer progression, effected via the dynamics of GRN and PPI networks in cancer progression—all remain to be fully characterized.

Figure 2 shows a simple model for how the evolution of p53 and its paralogs may affect GRN topology; such molecular evolutionary information-asymmetric signaling games approaches may help to better understand the motifs associated with oncogenes in GRNs. An additional important factor in cancer is the pervasive occurrence of molecular deception (Bhatia and Kumar, 2013). From a signaling games perspective, the use of deception is consistent with cancer's conflict of interest with somatic cells. The identity of deceptive macromolecular signals may be incorporated into the network, potentially shedding a novel light on the mechanism of carcinogenesis. The genesis of deceptive signals therefore is expected to impact and drive carcinogenesis, with the level of deception increasing as the cancer progresses, and as its conflict with the soma intensifies. Of interest is the question whether there is an identifiable phase transition in network topology associated with metastasis. Taming this deception should therefore constitute a key counter-strategy in combating cancer, and is currently represented by the use of immunotherapy approaches (Zhang and Chen, 2018), although the game theoretical underpinning of these techniques has not been appreciated.

An additional factor to understanding this biology are copy number variants (CNVs)—types of gene mutations where a number of large sections of genomic DNA may be duplicated (or deleted), resulting in dosage effects of the resident gene sequences, which are exactly duplicated (or deleted). The numbers of CNVs can commonly vary substantially within a population, and have been shown to have significant roles in the propensity to develop cancer (Krepischi et al., 2012). An increase in the number of CNVs would have the effect of enhancing the weight of an edge, which represents the interaction of the CNV gene product with its macromolecular binding partner. Such a network variant represents an increased disposition to develop cancer, and can be understood as occupying a position in “network space” (the space of all possible network topologies) in greater proximity to an aberrant network, than a normal network.

**Problem 4.H** *Study Cancer progression models in terms of GRN's and identify the role of driver and passenger genes in the somatically evolving networks, and the number and distribution of deceptive signals*.

### 4.4. Gene Regulation and 3D Networks

The origin and development of GRNs from a signaling games perspective is discussed in the Introduction. However, GRNs typically do not take into account 3D spatial orientation, and this provides a more complete view of gene regulation. Recent work has outlined the importance of three-dimensional proximity of genes to genes on other chromosomes, in addition to their immediate neighborhood on their own chromosome (Li et al., 2018). This effect implies that gene proximity and spatial relationships within the nucleus can be meaningfully represented as a network. Such a network would be comprised of two types of edge: (1) linear distance on the same chromosome (centimorgans), (2) physical distance with genes on other chromosomes (nanometers). Such networks may be termed “3D gene orientation networks.”

Gene regulation and co-regulation may be better understood by the construction and analysis of 3D gene orientation networks. This is because the proximity of regulatory modules to a gene has an influence on gene expression. Most genes have a regulatory region 5′ of the transcription start site, the promoter. In addition, regulatory enhancers and other regulatory elements may be located distant from the gene, generally on the same chromosome (Gondor and Ohlsson, 2018). It is thought that the bending and juxtaposition of chromosomes within the nucleus may bring such elements into physical proximity to the gene (Gondor and Ohlsson, 2018). Clearly, the physical distance, and frequency with which the element is brought into contact with the gene will influence the nature of its regulatory input. Using 3D gene orientation networks, additional information may be incorporated into edges, such as whether physical proximity is static, or has movement. If there is movement, this may be coordinated (or not) with other regulatory elements affecting the same gene. Likewise, interactions with regulatory elements may show some coordination between genes. A signaling games aspect is incorporated by considering the regulatory elements as signals, the gene that is regulated as the sender, and DNA binding proteins that bind to the regulatory elements as receiver molecules, this scheme is illustrated in Figure 2.

**Problem 4.I** *Describe the Gene Duplication process and their signaling game utilities in terms of the genome's 3D structure*.

### 4.5. Generalization of Genetic Variations

This paper describes an idealized picture: it describes a canonical gene regulation network and variations affecting the associated (single) genome, among which gene duplication has taken a lion's share of the focus. This picture needs to be generalized to consider an ensemble of genomes, and variations to the implied ensemble of genetic networks, which can vary based on additional intra-genome variations: e.g., horizontal gene transfer, reverse transcription and recombination, but also due to effects such as cell-fusion and endosymbiosis and effect of population sizes (e.g., in allelic fixation, for instance in sex chromosomes). Mathematically, the implied models of family of graphs would be significantly complex and may require theories from large networks and graph limits to understand the asymptotic properties. We leave these and associated algorithmic questions as topics of future research.

**Problem 4.J** *Adding genome duplication and fusion, gene transfer, gene conversion, endosymbiosis, sexual recombination, fixation etc. to describe evolution of an ensemble of GRNs*.

## 5. Conclusion

Here, we have outlined graph theoretical approaches that may reveal some novel aspects of the molecular evolutionary process, incorporating the understudied factor of information asymmetry, whose effect may become manifest at the level of the phenome. Further work is required to link the diverse features of network topology with network evolution and growth. While the evolutionary aspects shaping individual gene-gene interactions has been addressed by geneticists and molecular evolutionists, we believe that a synthesis entailing a multi-disciplinary effort combining game theory, graph theory, and algebraic/statistical analysis will provide a more informative omnigenic model of gene interactions, in contrast to the traditional homogenic view. Given our view that biomolecular networks may be modeled using evolutionary game theory, and given that evolutionary game theoretical approaches have been used in the study of social networks, we expect that some surprising similarities and convergences between the topologies of the two might be observed. Finally, we note that the field of statistics gained impetus from the consideration of biological problems, from workers such as Fisher, Haldane, Rao, Wright, Kimura, Crow, and others, and so we suggest that consideration of the open problems listed here might also lead to a similar development of new mathematics.

## 6. Bibliographic Notes

We recommend the following articles for further reading: (Albert and Barabási, 2002; Barabási et al., 2002, 2003, 2004; Farkas et al., 2002; Schwartz et al., 2002; Barabási, 2003; Chung and Lu, 2004, 2006; Candia et al., 2008; Goh and Barabási, 2008; Vazquez et al., 2008; Davis et al., 2010; Song et al., 2010; Liu et al., 2013; Janwa and Rangachari, 2015). For other important sources (especially with respect to directed graphs), we refer to Newman and Watts (1999), Newman (2001, 2003b,c,d, 2004, 2006, 2010), Girvan and Newman (2002), Meyers et al. (2006), Moore et al. (2006), Clauset et al. (2009), Karrer and Newman (2010), Newman et al. (2011), Zhang et al. (2016, 2017). For evolution of networks (see for example Sharan et al., 2005; Mazurie et al., 2010). For bipartite networks (Janwa and Lal, 2003; Høholdt and Janwa, 2012). For Spectral methods (Cvetković et al., 1980; Lubotzky et al., 1988; Lubotzky, 1994, 2012; Chung, 1997; Davidoff et al., 2003; Sarnak, 2004; Chung and Lu, 2006; Spielman and Teng, 2011; Janwa and Rangachari, 2015).

## Author Contributions

BM conceived of and structured the presented ideas at a high level. SM and BM developed the biological theories. HJ, BM, and JV developed the computational, quantitative, and mathematical theories. All authors discussed the open problems and contributed to the final manuscript.

### Conflict of Interest Statement

The authors declare that the research was conducted in the absence of any commercial or financial relationships that could be construed as a potential conflict of interest.

## Footnotes

^1^A mutation in MDM affects all p53, p63, and p73 allowing utility tradeoffs between fecundity (through decreased embryonic lethality) and cancer risks (through reduced somatic surveillance) in a population.
