# Supplementary material for: On the Origin of Biomolecular Networks
Source: Front Genet. 2019 Apr 10;10:240. doi: 10.3389/fgene.2019.00240 (PMC6467946; doi:10.3389/fgene.2019.00240)
Supplement: Supplementary file 1 [file Table_1.pdf]

Figure3–Luke–edgelist

Andrew	Bartholomew
Andrew	James
Andrew	JamesAlphaeus
Andrew	John
Andrew	JudasIscariot
Andrew	Levi–Matthew
Andrew	Phillip
Andrew	Simon
Andrew	Thaddeus
Andrew	Thomas
Andrew	Jesus
Andrew	Peter
Andrew	Joanna
Andrew	Cleopas
Andrew	MaryMagdalene
Andrew	MaryMotherJames
Bartholomew	James
Bartholomew	JamesAlphaeus
Bartholomew	John
Bartholomew	JudasIscariot
Bartholomew	Levi–Matthew
Bartholomew	Phillip
Bartholomew	Simon
Bartholomew	Thaddeus
Bartholomew	Thomas
Bartholomew	Jesus
Bartholomew	Peter
Bartholomew	Joanna
Bartholomew	Cleopas
Bartholomew	MaryMagdalene
Bartholomew	MaryMotherJames
James	JamesAlphaeus
James	John
James	JudasIscariot
James	Levi–Matthew
James	Phillip
James	Simon
James	Thaddeus
James	Thomas
James	Jesus
James	Peter
James	Joanna
James	Cleopas
James	Elijah
James	Moses
James	God
James	Jairus

James	JairusWife
James	Zebedee
James	MaryMagdalene
James	MaryMotherJames
JamesAlphaeus	John
JamesAlphaeus	JudasIscariot
JamesAlphaeus	Levi–Matthew
JamesAlphaeus	Phillip
JamesAlphaeus	Simon
JamesAlphaeus	Thaddeus
JamesAlphaeus	Jesus
JamesAlphaeus	Peter
JamesAlphaeus	Joanna
JamesAlphaeus	Cleopas
JamesAlphaeus	Alphaeus1
JamesAlphaeus	MaryMagdalene
JamesAlphaeus	MaryMotherJames
John	JudasIscariot
John	Levi–Matthew
John	Phillip
John	Simon
John	Thaddeus
John	Thomas
John	Jesus
John	Peter
John	Joanna
John	Cleopas
John	Elijah
John	Moses
John	God
John	Jairus
John	JairusWife
John	Zebedee
John	MaryMagdalene
John	ManEarthenwareJar
John	MaryMotherJames
JudasIscariot	Levi–Matthew
JudasIscariot	Phillip
JudasIscariot	Simon
JudasIscariot	Thaddeus
JudasIscariot	Thomas
JudasIscariot	Jesus
JudasIscariot	Peter
JudasIscariot	Joanna
JudasIscariot	Cleopas
JudasIscariot	MaryMagdalene
JudasIscariot	MaryMotherJames
Levi–Matthew	Phillip
Levi–Matthew	Simon
Levi–Matthew	Thaddeus

Levi–Matthew	Thomas
Levi–Matthew	Jesus
Levi–Matthew	Peter
Levi–Matthew	Joanna
Levi–Matthew	Cleopas
Levi–Matthew	MaryMagdalene
Levi–Matthew	MaryMotherJames
Phillip	Simon
Phillip	Thaddeus
Phillip	Thomas
Phillip	Jesus
Phillip	Peter
Phillip	Joanna
Phillip	Cleopas
Phillip	MaryMagdalene
Phillip	MaryMotherJames
Simon	Thaddeus
Simon	Thomas
Simon	Jesus
Simon	Peter
Simon	Joanna
Simon	Cleopas
Simon	MaryMagdalene
Simon	MaryMotherJames
Thaddeus	Thomas
Thaddeus	Jesus
Thaddeus	Peter
Thaddeus	Joanna
Thaddeus	Cleopas
Thaddeus	MaryMagdalene
Thaddeus	MaryMotherJames
Thaddeus	JamesFatherThaddeus
Thomas	Jesus
Thomas	Peter
Thomas	Joanna
Thomas	Cleopas
Thomas	MaryMagdalene
Thomas	MaryMotherJames
Angel	Jesus
Jesus	Anna
Jesus	Peter
Jesus	BleedingWoman
Jesus	BlindManSideRoad1
Jesus	CaiaphasSlave
Jesus	Centurion
Jesus	Herod
Jesus	Joanna
Jesus	Cleopas
Jesus	DemonPossessedManTomb1
Jesus	DinnerGuest

Jesus	Elijah
Jesus	Moses
Jesus	JohnBaptist
Jesus	Mary
Jesus	Pilate
Jesus	Jairus
Jesus	JairusDaughter
Jesus	JairusRepresentative
Jesus	Leper
Jesus	ManUncleanSpirit
Jesus	ManinCrowd
Jesus	OnlySonWidowfromNain
Jesus	Satan
Jesus	WidowfromNain
Jesus	YoungChild
Jesus	MaryMagdalene
Jesus	Joseph
Jesus	JosephArimathea
Jesus	KnownSinner
Jesus	ManDropsy
Jesus	ManVersedinLaw
Jesus	ManVersedinLaw2
Jesus	ManWitheredHand
Jesus	SonManinCrowd
Jesus	ManinCrowd2
Jesus	ManinCrowd3
Jesus	ManinCrowd4
Jesus	ManinCrowd5
Jesus	Martha
Jesus	ParalyzedMan
Jesus	PetersMotherinLaw
Jesus	PhariseeDinner
Jesus	PhariseeLeader
Jesus	PoorWomanTemple
Jesus	PresidingOfficer
Jesus	Robber1
Jesus	Robber2
Jesus	Simeon
Jesus	SimonCyrene
Jesus	SimonPharisee
Jesus	SpeechlessMan
Jesus	Susanna
Jesus	WeakWoman
Jesus	WomaninCrowd
Jesus	YoungMan
Jesus	Zacchaeus
Anna	Phanuel
AnotherPerson	Peter
Peter	Joanna
Peter	Cleopas

Peter	Elijah
Peter	Moses
Peter	God
Peter	Jairus
Peter	JairusWife
Peter	MaryMagdalene
Peter	ManEarthenwareJar
Peter	MaryMotherJames
Peter	PetersMotherinLaw
Peter	ServantGirl
Caiaphas	CaiaphasSlave
CaiaphasSlave	DisciplewithSword
Chuza	Herod
Chuza	Joanna
Herod	JohnBaptist
Herod	PhillipHerodsBrother
Herod	Pilate
Herod	Herodias
Herod	Lysanius
Joanna	ManShiningGarment1
Joanna	ManShiningGarment2
Joanna	MaryMotherJamesJoses
Joanna	MaryMagdalene
Elijah	Moses
Elijah	WidowZarephath
Elizabeth	JohnBaptist
Elizabeth	Mary
Elizabeth	Zechariah
JohnBaptist	Zechariah
Mary	Gabriel
Mary	Zechariah
Mary	Joseph
Mary	Simeon
Gabriel	Zechariah
Gabriel	God
PhillipHerodsBrother	Pilate
PhillipHerodsBrother	Herodias
PhillipHerodsBrother	Lysanius
Pilate	JosephArimathea
Pilate	Lysanius
Pilate	Barabbas
Jairus	JairusDaughter
Jairus	JairusWife
Jairus	JairusRepresentative
JairusDaughter	JairusWife
ManinCrowd	SonManinCrowd
OnlySonWidowfromNain	WidowfromNain
Satan	Judas
ManShiningGarment1	ManShiningGarment2
ManShiningGarment1	MaryMotherJamesJoses

ManShiningGarment1 MaryMagdalene
ManShiningGarment2 MaryMotherJamesJoses
ManShiningGarment2 MaryMagdalene
MaryMotherJamesJoses MaryMagdalene
MaryMotherJamesJoses JamesLess
Joseph Simeon
KnownSinner SimonPharisee
ManinCrowd3 DeadFather
Martha MarySisterMartha
Melchi Neri
Officer OfficersSlave
Robber1 Robber2
