# Supplementary material for: On the Origin of Biomolecular Networks
Source: Front Genet. 2019 Apr 10;10:240. doi: 10.3389/fgene.2019.00240 (PMC6467946; doi:10.3389/fgene.2019.00240)
Supplement: Supplementary file 2 [file Table_2.pdf]

Figur33–Mathew–edgelist

Joseph	Mary
Mary	Jesus
GodsAngel	Joseph
God	Mary
God	Isaiah
Joseph	Jesus
God	Hosea
God	Jeremiah
Archelaus	HerodGreat
JohnBaptist	Jesus
JohnBaptist	God
JohnBaptist	Isaiah
Jesus	God
Jesus	Satan
Jesus	Peter
Jesus	Andrew
Peter	Andrew
Jesus	James
Jesus	John
James	Zebedee
John	Zebedee
James	John
Jesus	Leper
Jesus	Officer
Officer	OfficersSlave
Jesus	PetersMotherinLaw
Peter	PetersMotherinLaw
CapernaumScribe	Jesus
DemonPossessedManTomb1	Jesus
DemonPossessedManTomb2	Jesus
DemonPossessedManTomb1	DemonPossessedManTomb2
ParalyzedMan	Jesus
Jesus	Levi–Matthew
Jairus	Jesus
Jairus	JairusDaughter
Jesus	JairusDaughter
Jesus	BleedingWoman
Jesus	BlindMan1
Jesus	BlindMan2
BlindMan1	BlindMan2
Jesus	SpeechlessMan
Jesus	Phillip
Jesus	Bartholomew
Jesus	Thomas
Jesus	JamesAlphaeus
Jesus	Thaddeus

Jesus	Simon
Jesus	JudasIscariot
JamesAlphaeus	Alphaeus1
Peter	James
Peter	John
Peter	Phillip
Peter	Bartholomew
Peter	Thomas
Peter	Levi–Matthew
Peter	JamesAlphaeus
Peter	Thaddeus
Peter	Simon
Peter	JudasIscariot
Andrew	James
Andrew	John
Andrew	Phillip
Andrew	Bartholomew
Andrew	Thomas
Andrew	Levi–Matthew
Andrew	JamesAlphaeus
Andrew	Thaddeus
Andrew	Simon
Andrew	JudasIscariot
James	Phillip
James	Bartholomew
James	Thomas
James	Levi–Matthew
James	JamesAlphaeus
James	Thaddeus
James	Simon
James	JudasIscariot
John	Phillip
John	Bartholomew
John	Thomas
John	Levi–Matthew
John	JamesAlphaeus
John	Thaddeus
John	Simon
John	JudasIscariot
Phillip	Bartholomew
Phillip	Thomas
Phillip	Levi–Matthew
Phillip	JamesAlphaeus
Phillip	Thaddeus
Phillip	Simon
Phillip	JudasIscariot
Bartholomew	Thomas
Bartholomew	Levi–Matthew
Bartholomew	JamesAlphaeus
Bartholomew	Thaddeus

Bartholomew	Simon
Bartholomew	JudasIscariot
Thomas	Levi–Matthew
Thomas	JamesAlphaeus
Thomas	Thaddeus
Thomas	Simon
Thomas	JudasIscariot
Levi–Matthew	JamesAlphaeus
Levi–Matthew	Thaddeus
Levi–Matthew	Simon
Levi–Matthew	JudasIscariot
Thaddeus	Simon
Thaddeus	JudasIscariot
Simon	JudasIscariot
Jesus	ManWitheredHand
Jesus	JamesJust
Jesus	JosephBrother
Jesus	SimonBrother
Jesus	JudasBrother
JamesJust	Mary
JosephBrother	Mary
SimonBrother	Mary
JudasBrother	Mary
Herod	JohnBaptist
Herod	PhillipHerodsBrother
Herodias	PhillipHerodsBrother
Herodias	JohnBaptist
Herodias	Herod
Herodias	HerodiasDaughter
HerodiasDaughter	JohnBaptist
Jesus	PhoenecianWoman
Jesus	PhoenecianWomansDaughter
PhoenecianWoman	PhoenecianWomansDaughter
Peter	Jonah
Moses	Peter
Moses	James
Moses	John
Elijah	Peter
Elijah	James
Elijah	John
Moses	Elijah
Moses	Jesus
Elijah	Jesus
God	Peter
God	James
God	John
Jesus	FatherEpilepticSon
FatherEpilepticSon	EpilepticSon
Jesus	EpilepticSon
Jesus	YoungChild

YoungMan	Jesus
MotherSonsZebedee	James
MotherSonsZebedee	John
MotherSonsZebedee	Zebedee
MotherSonsZebedee	Jesus
Jesus	BlindManSideRoad1
Jesus	BlindManSideRoad2
ManVersedinLaw	Jesus
ZechariahProphet	Barachiah
Jesus	SimonLeper
WomanwithJar	Jesus
DisciplewithSword	CaiaphasSlave
CaiaphasSlave	Caiaphas
Jesus	DisciplewithSword
Jesus	Caiaphas
FalseWitness1	Caiaphas
FalseWitness2	Caiaphas
FalseWitness1	Jesus
FalseWitness2	Jesus
ServantGirl	Peter
Pilate	Jesus
Pilate	PilatesWife
PilatesWife	Jesus
Pilate	Barabbas
Jesus	SimonCyrene
Robber1	Jesus
Robber2	Jesus
Bystander	Jesus
Jesus	Centurion
MaryMagdalene	Jesus
MaryMotherJamesJoses	JamesLess
MaryMotherJamesJoses	Joses
MaryMotherJamesJoses	Jesus
MaryMagdalene	MaryMotherJamesJoses
MaryMotherJamesJoses	MotherSonsZebedee
MaryMagdalene	MotherSonsZebedee
GodsAngel	MaryMagdalene
GodsAngel	MaryMotherJamesJoses
JosephArimathea	Pilate
JosephArimathea	Jesus
