# Supplementary material for: On the Origin of Biomolecular Networks
Source: Front Genet. 2019 Apr 10;10:240. doi: 10.3389/fgene.2019.00240 (PMC6467946; doi:10.3389/fgene.2019.00240)
Supplement: Supplementary file 3 [file Data_Sheet_1.PDF]

Supplementary Material: On the Origin of Biomolecular Networks

1 GRAPH THEORY AND ITS APPLICATION TO BIOMOLECULAR NETWORKS

In this section, we discuss fundamentals of graphs and networks as well as other important topics that are critical for the study and analysis of biomolecular networks.

Our discussions deal with an abstraction that facilitates reasoning about a set of entities, denoted V and a binary relation $E \subseteq V \times V$: the binary relation is usually irreflexive, asymmetric and not necessarily transitive. It is often represented as a directed graph, with vertices V and edges E. When V denotes biomolecules and E denotes interactions (e.g., regulations, proximity, binding, synteny, etc.), the resulting graph is called a biomolecular network, object of our study. Such networks evolve over time with additions and deletions to the sets V and E.

GRAPHS FROM A COMBINATORIAL PERSPECTIVE

We collect here essential results from graph theory. For these results, we refer to Serre Serre (1980) and Biggs Biggs (1993).

DEFINITION 1. A (directed) graph X is a pair of sets $\mathbb{X} = (V, E)$ (the set of vertices V and edges E respectively)*, where a directed edge e is defined as an ordered pair of vertices $e = (a, b)$, where a is the origin of the directed edge e and b is the terminus of e. A rigorous definition then is augmented with two maps*

$$\phi_1 : \quad E \to V \times V : \qquad e \mapsto (o(e), t(e)),$$

and

$$\phi_2 : \; E \to E : \qquad e \mapsto \overline{e}$$

that satisfy the following condition: for each e, $\overline{\overline{e}} = e$, $\overline{e} \neq e$, and $t(\overline{e}) = o(e)$. The bar operation is reversing the direction. Henceforth, we denote the graph $X = (V, E, o, e)$ as a 4-tuple.

For each edge $e \in E$, $o(e)$ is called the *origin* of the edge e and $t(e)$ is called the *terminus* of the edge e. A graph $Y = (V', E', o', t')$ is called a subgraph of X if $V' \subseteq V$, $E' \subseteq E$ and o' and t' are restrictions of o and t respectively to E'.

For some studies we need to provide an orientation of an undirected graph. An *orientation* of a graph X is a subset E_+ of the edges such that E is the disjoint union of E_+ and $\overline{E_+}$.

DEFINITION 2. A *walk* of length n in a graph is a sequence of alternating vertices and edges,

$$\langle v_0, e_1, v_1, e_2, \ldots, v_n, e_n \rangle,$$

such that $o(e_i) = v_{i-1}$ and $t(e_i) = v_i$ for all $i = 1, \ldots, n$.

DEFINITION 3. A graph $X = (V, E, o, e)$ is said to be connected if any two vertices are the extremities of at least one walk. The maximally connected subgraphs (under the relation of inclusion) are called the connected components of X.

DEFINITION 4. A graph is called *bipartite* if the vertex set can be partitioned into two parts V_1 and V_2 such that each edge has one vertex in V_1 and one vertex in V_2.

The distance between two vertices u and v is the length of the shortest walk connecting them, if both vertices are in the same connected component (∞, otherwise). The shortest walk connecting u and v is called a *geodesic*.

Let n be an integer ≥ 1. Consider the oriented graph on Z/nZ, and the orientation is given by the edges $[i, i+1]$ ($i \in Z/nZ$) with $o([i, i+1]) = i$ and $t([i, i+1]) = i+1$.

DEFINITION 5. A subgraph Y of a graph X is called a *circuit* of length n if it is isomorphic to the circle graph on Z/nZ.

A circuit of length 1 is called a *loop*. If the relation E is irreflexive then the graph is loop-free.

DEFINITION 6. A graph is called *combinatorial or simple* if it has no circuit of length ≤ 2.

DEFINITION 7. A non-empty connected graph T without circuits is called a tree.

DEFINITION 8. A weighted graph G has weights assigned with edges, by the weight function, $w : V \times V \to \mathbb{R}$ non-negative, with $w(u, v) = 0$ if and only if $e = (u, v) \notin E$. The weighted degree d_v of a vertex v is defined as $d_v := \sum_u w(v, u)$. We also define volume V of the graph as $vol(G) := \sum_v d_v$.

GRAPHS FROM AN ALGEBRAIC PERSPECTIVE

Certain linear operators can be associated with a graph and can be given a physical meaning in terms of diffusion (of information) over the graph, as common in the signaling games over the biomolecular networks. Spectral analysis of such linear operators yields eigenvalues, eigenvectors, and spectra of graphs, playing important roles in determining various properties of the network – specifically, with respect to how information diffuses over them (see Chung (1997) and Chung and Lu (2006)).

DEFINITION 9. Algebraically a graph G (network) can be represented as an $n \times n$ adjacency matrix $A(G)$, in which, A_{ij} is 1 iff $\exists e \in E, o(e) = i \ \& \ t(e) = j$; otherwise it is 0. The matrix is symmetric if the graph is undirected, i.e., $e = \bar{e}, \forall e \in E$. If the graph G is weighted, then $A_{ij} = w(i, j)$ for every edge $(i, j) = e \in E$, and 0, otherwise.

We can think of A as operating on the space $V = C^n$ of complex n-tuples written as column vectors X as follows: $X \to AX$. X can be thought of as values of a function evaluated on the vertices. One can show that there exist lines through the origin, in V that are left invariant along those lines. That is to say, there exist scalars λ_i (called eigenvalues), and corresponding non-zero vectors X_i (called eigenvectors spanning invariant lines) that span invariant lines such that $A_i = \lambda_i X$, for $1 \leq i \leq n$. The *spectrum* of the graph X is defined to be $Spec(X) := Spec(A) := \{\lambda_1, \cdots, \lambda_n\}$, a collection of A's eigenvalues.

It can also be shown that if A is a real symmetric matrix, then the eigenvalues of A are real and its spectrum can be presented in decreasing order, i.e., $\{\lambda_1 \geq \lambda_2 \geq \cdots \lambda_n\}$. This fact is very important for our study of graphs and networks.

Let us consider a more general weighted graph as defined earlier. Let T be the diagonal matrix with d_v along the diagonal. First, consider the stochastic matrix $P = T^{-1}A$, which may be thought of as describing the probabilities of certain "information" being moved from one node to a neighboring node by a diffusion process. Let $\{v_0, e_0, v_1, e_1, \cdots, v_s\}$ be a random walk in the graph with $(v_{i-1}, v_i) \in E(G)$,

for all $1 \leq i \leq s$, and determined by transition probabilities $P(u, v) = Prob(x_{i+1} = v | x_i = u)$ which are independent of i. Normally we take $p(u, v) = w(u, v)/d_u$, as defined by the stochastic matrix P.

Then, let $f : V \to \mathbb{R}$ with $\sum_v f(v) = 1$ be a probability distribution on $V(G)$. Then $\sum_v P(u, v) = 1$. Then for any initial distribution $f : V \to \mathbb{R}$ with $\sum_v f(v) = 1$, the distribution after k steps is $P^k f$, where f is viewed as a column vector and P is the matrix of transition probabilities. In particular, a probability distribution satisfying the fixed point equation $\phi = P\phi = P^2\phi = \cdots = P^k\phi$ describes the stationary distribution of the diffusion process and can be described as an eigenvector of the corresponding matrix.

Thus intuitively, algebraic techniques allow thinking about the graph features in terms of a set of "blurrier" notions such as random walks (instead of walks), diffusion distances (instead of geodesic distances), ranks (instead of informational relevance), etc. However, because such spectral analysis is based on linear algebra, the underlying algorithms become tractable.

The adjacency matrix should be best viewed as an operator on functions of $V(G)$. A modified operator, called the Laplacian operator is the most effective formulation. The Laplacian operator can be used in interpolation on graphs, graph clustering, resistance networks, rapid mixing, linear solving, linear optimization, and many other applications.

Thus, one may define $L = T - A = T(I - P)$, as the *Laplacian Matrix* of G, where L is defined as follows: $L(u, v) = -w(u, v)$ (when u and v are distinct), and d_v if $u = v$. Imagine assigning a scalar-valued *rank* function $\rho : V(G) \to \mathbb{R} : v \mapsto \rho(v)$ such that the following *Dirichlet Sum* of G

$$\sum_{(u,v) \in E} w(u, v) \left(\rho(u) - \rho(v)\right)^2,$$

is minimized. Thus ρ has the meaning that if a gene in a GRN is important then the genes it regulates and the genes that regulate it are also important; one expects p53 to be labeled as an important gene because of its "hubbiness," but so also, MDM1, ATM, BRCA1, etc. as they are in the pathways directly regulating p53; and also making the genes such as p63 and p73 important as they are regulated by this cluster of genes (which may have preferentially attached themselves to p53 and its duplicates, which they continue to regulate). Note that solution to the optimization problem for Dirichlet sum is given by the following equation (under suitable conditions—see Gleich (2015); Easley and Kleinberg (2010); Chung (1997); Chung and Lu (2006)):

$$\frac{1}{d(x)} \sum_{(y,x) \in E} (\rho(x) - w(y, x)\rho(y)) = (I - P)\rho = 0.$$

Thus functions such as ρ can be rapidly computed by iterating over the graph while performing weighted-averaging. An example of this process is seen in Google's PageRank algorithm based on the "Random Surfer (with Teleportation) Model,". This and other PageRank algorithms have been successfully applied *mutatis mutandis* to rank genes in a GRN (GeneRank), to rank Proteins in a PPI Networks (PPIRank), and in other biomolecular networks (see the survey Gleich (2015)).

From now, we assume that G is weight symmetric $w(u, v) = w(v, u)$. Then the eigenvalues of $L(G)$ are real, and indeed $0 = \lambda_0 \leq \lambda_1 \leq \cdots \leq \lambda_{n-1}$. Then $\lambda_G := \lambda_1$ is called the *spectral gap* of G. The spectral gap (and other eigenvalues) can be determined by the Courant-Fisher theorem. For example, if one

considers L as an operator on the space of functions $g : V(G) \to \mathbb{R}$, then

$$\lambda_G := \lambda_1 = \inf_{g \perp \mathbf{1}} \frac{\langle g, L \rangle}{\langle g, g \rangle}$$

One can show that if the spectral gap λ_G is large, and k is large enough any initial distribution f converges to the stationary distribution very rapidly.

EXPANSION PROPERTIES AND INFORMATION FLOWS IN GRAPHS

We shall consider graphs $X = (V, E, \cdot, \cdot)$, where V is the set of vertices and E is the set of edges of X. We will assume that the graph is undirected and connected and we shall only consider finite graphs. For $F \subset V$, the *boundary* ∂F is the set of edges connecting F to $V \setminus F$. The *expanding constant*, or *isoperimetric constant* of X is defined as,

$$h(X) = \min_{\emptyset \neq F \subset V} \frac{|\partial F|}{\min\{|F|, |V \setminus F|\}} \tag{S1}$$

Moreover if X is viewed as the graph of a communication network, then $h(X)$ measures the quality of the network as a transmission network. In all applications, the larger the $h(X)$ the better, so we seek graphs (or families of graphs) with $h(X)$ as large as possible with some fixed parameters.

In Tanner (1984), M. Tanner introduced another notation for the expansion coefficient. Let as before $X = (V, E, \cdot, \cdot)$, be a graph where V is the set of vertices and E is the set of edges of X. Let $X \subseteq V$ with $|X| \leq \alpha |V|$, then

$$c(\alpha) = \min_{\emptyset \neq X \subset V \wedge |X| \leq \alpha |V|} \frac{|\partial X|}{\min\{|X|, |V \setminus X|\}} \tag{S2}$$

It is well-known that the expansion properties of a graph are closely related to the eigenvalues of the *adjacency matrix* A of the graph $X = (V, E)$; it is indexed by pairs of vertices x, y of X and A_{xy} is the number of edges between x and y. When X has n vertices, A has n real eigenvalues, repeated according to multiplicities that we list in decreasing order

$$\lambda_0 \geq \lambda_1 \geq \ldots \geq \lambda_{n-1}.$$

It is also known that if X is D-regular, i.e. all vertices have degree D, then $\lambda_0 = D$ and if moreover the graph is connected $\lambda_1 < D$. Also X is bipartite if and only if $-\lambda_0$ is an eigenvalue of A. We recall the following (see for example Chung (1997) Davidoff et al. (2003)):

THEOREM 1. *Let X be a finite, connected, D-regular graph then*

$$(D - \lambda_1)/2 \leq h(X) \leq \sqrt{2D(D - \lambda_1)}.$$

And

THEOREM 2. *(see Chung (1997), Davidoff et al. (2003)) Let $(X_m)_{m \geq 1}$ be a family of finite connected, D-regular graphs with $|V_m| \to +\infty$ as $m \to \infty$. Then*

$$\liminf_{N \to \infty} \lambda_1(X_m) \geq 2\sqrt{D - 1}.$$

This leads to the following.

DEFINITION 10. *A finite connected, D-regular graph X is* Ramanujan *if, for every eigenvalue λ of A other than $\pm D$, one has $\lambda \leq 2\sqrt{D-1}$.*

We will also need an important definition.

DEFINITION 11 (Bipartite Ramanujan Graphs). *Let X be a (c, d)-regular bipartite graph. Then X is called a Ramanujan graph if*

$$\lambda(X) \leq \sqrt{(c-1)} + \sqrt{(d-1)}.$$

It is known that computing the expansion coefficient of arbitrary graphs is an NP-complete problem. Thanks to the work of Tanner, and Alon and Millman, one can derive bounds on the expansion coefficient in terms of λ. The complexity of determining λ, though in P, is still difficult if the number of vertices is large (for example of the order 10^4-10^6 or more for biomolecular networks such as GRN or PPI).

There are useful bounds on λ for arbitrary (bipartite) graph X in terms of the number of edges, the maximum degree λ_{max}, and the rank r_χ of the adjacency matrix of X. Since effective upper bounds exist on $\lambda_{\max}$, and r_χ, (see Hø holdt and Janwa (2012)) we thus obtain a bound that is easily computable.

An expander graph is a highly connected sparse graph (see, for example Sarnak (2004)). Expander graphs have numerous applications including those in communication science, computer science (especially complexity theory), network design, cryptography, combinatorics and pure mathematics (see the references under Bibliographic Notes below))). Expander graphs have played a prominent role in recent developments in coding theory (LDPC codes, expander codes, linear time encodable and decodable codes, codes attaining the Zyablov bound with low complexity of decoding (see the Bibliographic notes for references).

DEFINITION 12. A matrix A with rows and columns indexed by a set X is called irreducible when it is not possible to find a proper subset S of X so that $A(x, y) = 0$, whenever $x \in S$ and $y \in X \setminus S$. Equivalently, A is not irreducible if and only if it is possible to apply a simultaneous row and column permutation on A to get a matrix in a square block form so that one of the blocks is a zero block. For the following lemma, see for example (Horn and Johnson (2013) p. 363).

LEMMA 1. *Let D be a finite graph. Then the adjacency matrix of A is irreducible if and only if D is connected.*

We shall also need the following.

PROPOSITION 1 (Perron-Frobenius). *Let A be an irreducible non-negative matrix. Then, there is up to scalar multiples, a unique non-negative eigenvector $\mathbf{a} := (a_1, a_2, \cdots, a_n)$ all of whose coordinates a_i are strictly positive. The corresponding eigenvalue λ_0 (called the dominant eigenvalue of A) has algebraic multiplicity 1 and $\lambda_0 \geq \lambda_i$ for any eigenvalue λ_i of A.*

We recall the following special case of Courant-Fisher theorem (also, called the Raleigh-Ritz Theorem) (see for example, (Horn and Johnson (2013), Theorem 4.2.2))

THEOREM 3. *Let A be an $n \times n$ Hermitian matrix over the complex field C, then it is known that all its eigenvalues are real, with maximum eigenvalue $\lambda_{\max}$ (i.e. the spectral radius of A). For $\mathbf{0} \neq X \in C^n$,*

define the Raleigh quotient

$$R_X := \frac{X^{*T}AX}{X^{*T}X}.$$

Then $\lambda_{\max} = \max_{X \neq \mathbf{0}} R_X$*. Furthermore,* $R_X \leq \lambda_{\max}$ *with equality if and only if* X *is an eigenvector corresponding to the eigenvalue* $\lambda_{\max}$*.*

GROUPS ACTING ON GRAPHS

To understand symmetries of graphs, and to understand original motivation of graphs as objects associated with topology and algebraic topology, we briefly discuss groups acting on graphs (as groups acting on topological spaces). This converts graph isomorphism problems into much more manageable group isomorphism problem. It is needed in alignment and motif detection, We say that a group G *acts* on a graph $X(V, E, o, t)$ if it acts on V and E such that the actions are compatible with ϕ_1 and ϕ_2, i.e., it commutes with ϕ_1 and ϕ_2, i.e., $g(\phi_1(e)) = \phi_1(g(e))$, $o(g(v)) = g(o(v))$, $t(g(v)) = g(t(v))$ and $g(\phi_2(e)) = \phi_2(g(e))$, $\forall g \in G$.

An *inversion* is a pair consisting of an element $g \in G$ and an edge $e \in E$ such that $g(e) = \overline{e}$. We will say that a group acts *freely* on X if it acts without inversion and $g = 1$ is the only element having a fixed point. For the following result, we refer to Serre (Serre, 1980, Page 27).

THEOREM 4. *If G acts freely on a tree, then G is a free group.*

DEFINITION 13. *The Graph $X(G, S)$*: Let G be a group and $S \subseteq G$. The *Cayley graph* $X(G, S)$ is defined as the oriented graph with the vertex set G and edge set $E = G \times S$.

$$o(g, s) = g \quad \text{and} \quad t(g, s) = gs$$

The group G acts on $X(G, S)$ by left multiplication. This action preserves orientation; and its action is free on the set of vertices and on the set of edges.

PROPOSITION 2. *i) $X(G, S)$ is connected if and only if S generates G. In fact, the connected components correspond in a 1-1 fashion to the cosets of $H = \langle S \rangle$ (the group generated by S).*

ii) X contains a loop if and only if $1 \in S$.

iii) For X to be combinatorial, it is necessary and sufficient that $S \cap S^{-1} = \emptyset$.

The adjacency matrix provides significant amount of information about the graph. For example A^r gives the number of walks of length r between vertices.

DEFINITION 14. The *girth* of X is the smallest positive integer r such that $Trace(A^r) > 0$. Let $d(X)$ be the smallest integer (if it exists) so that for every pair of vertices (u, v) there is a walk of length at most d from u to v. Then $d(X)$ is called the *diameter* of the graph X.

Important results about diameter, girth and other combinatorial invariants (important for biomolecular networks) and bounds on them in terms of spectral invariants can be found for general graphs in Bollobas (Bollobás (1978), pp. 156–157).

LEMMA 2. *Let X be a k-regular graph. Then*

i) $\lambda_i \leq k$, *for* $0 \leq i \leq n-1$.

ii) $\lambda_0 = k$ *and* $m(\lambda_0)$ *equals the number of connected components of* X.

iii) $\lambda_{n-1} = -k$ *if and only if* X *is bipartite.*

iv) For a bipartite graph X, *if* λ *is an eigenvalue with multiplicity* $m(\lambda)$, *then so is* $-\lambda$ *with multiplicity* $m(\lambda)$.

The adjacency matrix A can be considered as the matrix of a Hecke operator on $l^2(X)$ (which can be called the adjacency operator) as $A(f(x)) = \sum_{y \in V} A(x,y)f(y)$ (here $A(x,y) = 1$ if there is an edge from x to y and 0 otherwise). As mentioned earlier in the context of rank function, another interpretation of the adjacency operator is an averaging function of information contained on the neighboring vertices that flow along the adjacencies. An iterative process, then leads to mixing and diffusion globally in the network via the adjacency or the corresponding Laplacian operators.

MISCELLANEOUS RELATED COMMENTS

Remark (1)

The topics we expanded upon are the following (in order): (1) graphical representation of Networks; (2) algebraic representation of graphs; and finally, (3) algebraic properties, such as spectral analysis that provide us large number of tools and techniques to deduce various properties of the biomolecular networks. For complex and mid-size networks and models, there are important algorithmic questions related to random graphs and their evolution (Erdös-Renyi model), degree-distribution, power laws, preferential attachment models, scale free networks, random-walks and mixing, spectral distance, graph similarity, clustering, smoothing analysis, sparsification and linear solvers and applications.

Remark (2)

Algebraic representation of biomolecular networks has several advantages. In particular, the combinatorial meaning is inherent in the Adjacency matrix $A(X)$ of a graph X. In the study of biomolecular networks, it is very important to know the number of walks of length m between any two entities i to j, w_{ij}^m, (e.g. between any two proteins in the PPI network). But that is given by the $(ij)^{\text{th}}$ entry in A^m. From this one can deduce that the diameter of the network is the dimension of what is called the adjacency algebra $A(L)$ associated with A. If this diameter is small, we can deduce that the networks shows small world phenomenon, as we explained in the main text. The sequence (w_{ij}^m) can be put into coefficients of a series called the zeta function $\zeta(X)$ of the graph X, and it has a very simple form as a rational polynomial in terms of what are called spectra (or frequencies) of the network. And from $\zeta(X)$ we can find simple expressions for the set of walks.

Remark (3)

One can explain spectra (frequencies) of a biomolecular network in an intuitive manner as follows. In analogy with the physical sciences, if one considers the whole network as a space, one can think of its vibration modes, that is the frequencies (eigenvalue spectra) and their amplitudes (corresponding eigenvectors). One can indeed determine the shape of the the biomolecular network from its Spectra (in the manner of Marc Kac (Can one determine the shape of the drums? (from its spectra) Kac (1966)), and its analogy to networks and graphs Chung (1966). The advanced matrix of the biomolecular network can be then shown to take a simple diagonal form with respect to the basis of eigenvectors, and that leads to immense simplification of explanation of many phenomena associated with the biomolecular networks,

and plays important roles in the spectral algorithms and their complexity analysis. The coefficients of polynomials whose roots are the eigenvalues, carries information about the motifs at the nodes. The spectral gap $\lambda(X)$ (the difference between the two largest frequencies) of the biomolecular network determines expansion in the biomolecular network, meaning how fast spreading and mixing takes places between a set of nodes. How fast partitioning and cuts can be carried out—-important ingredient of many algorithms in biomolecular networks. In particular the spectral gap of the biomolecular network, $\lambda(X)$, gives lower bounds in terms of the combinatorial invariant, the Cheeger constant, as defined below. We will state isoperimetric inequality results where $\lambda(X)$ provides an excellent lower bound on $h(X)$, and for explanation of combinatorial phenomenon or for spectral approximation to combinatorial algorithms, $\lambda(X)$ can replace $h(X)$ and still one can get excellent approximate results. For example, it is very easy to show fast mixing and spreading phenomenon—-it is just $A^n f = \lambda(X)^n f$, where f is the initial distribution on the nodes.

Remark (4)

We have defined important algebraic concepts in graph theory in precise mathematical terms. Networks (molecular or otherwise) have algebraic representations as their adjacency matrix (from which one can re-derive the graphical representation if one wishes). From these one derives algebraic invariants such as eigenvalues and spectra of graphs, eigenvectors, and spectral gaps. In the main text we had defined the combinatorial invariant called the isoperimetric constant $h(G)$. The determination of $h(G)$ is an NP-complete problem. In the next sections we will show that $h(G)$ can be bounded in terms of the spectral gap $\lambda(X)$. This helps immensely in the determination of spectral graph algorithms as approximation to NP-complete combinatorial algorithms.

Remark (5)

One application of large spectral gap is rapid mixing (one can envisage that it is expected to prove very important in many biological applications). Indeed, the discrete analog of Cheeger inequality has increasingly crucial utility in the study of random walks and rapid mixing on Markov chains and new powerful spectral techniques such as Heat-Kernels and Sobolov inequalities have emerged to deal with general graphs (see Chung and Lu (2006), Chung and Lu (2004)). Rapid mixing in Markov chains can be framed as: How long does it take for an irreducible finite state Markov chain to converge to equilibrium? A fundamental application is to Markov Chain Monte Carlo (MCMC) simulation algorithms that are used widely in the scientific community to simulate Gibb's measures and to derive approximate solutions to difficult combinatorial questions. as Markov Chain Monte Carlo simulations are used widely in the scientific community to simulate Gibb's measures and to derive approximate solutions to difficult combinatorial questions—-that have high complexity and many of which appear in the topological analysis of biomolecular networks (such as clustering, community detection, and so on). Indeed two of the most heavily studied problems in the analysis of networks are graph clustering and graph diffusion. We already studied the importance graph clustering biomolecular networks in the main text. Graph diffusion refers to problems involving spreading or propagation along the edges of a graph. These problems are of fundamental important in algorithms such as PageRank and Hitts algorithms (see Easley and Kleinberg (2010), Levin and Peres (2017),Chung and Lu (2006), and Chung and Lu (2004)).

2 BIBLIOGRAPHIC NOTES

For further comprehensive treatment of applications of **spectral graph theory** to biomolecular networks we refer to Chung and Lu (2004) and the survey article Banerjee and Jost (2009). For state of

the art in spectral methods in algorithmic analysis, we refer to the lecture notes of Spielman (http://www.cs.yale.edu/homes/spielman) biomolecular networks algorithms in clustering, mixing, partitioning, random walks, Schur complements, effective resistance and applications, expander graphs and applications, graph sparsification and related algorithms, testing isomorphism of networks.

Related to spectral graph theory, **expander graphs** have become prominent in many recent developments in information and coding theory (LDPC codes, expander codes, linear time encodable and decodable codes, codes attaining the Zyablov bound with low complexity of decoding (see Tanner (1984), Sipser and Spielman (1996), Davidoff et al. (2003), Sarnak (1990), Lubotzky (1994), Hø holdt and Janwa (2012), Hø holdt and Janwal (2009), Janwa (2003), Janwa and Lal (2003), Janwa and Moreno (1998), Lubotzky (2012)),Sipser and Spielman (1996) Spielman (1996), Tanner (2001), Janwa and Lal (2003), Barg and Zémor (2004), Guruswami and Indyk (2001), and others).

The following articles are relevant for a comprehensive treatment of **spectral graph theory**, and **spectral graph theoretic algorithms** that are relevant to biomolecular networks described in the main text: Alon and Spencer (1992), Babai (1979), Bien (1989), Bollobás (1978), Bollobás (1986), Bollobás (1991), Brouwer et al. (1989), Chung (1991), Chung et al. (1988), Cvetković et al. (1980), Cvetković et al. (1988), Diaconis (1988), Diaconis (1991),Janwa and Rangachari (2015),Delgado and Janwa (2017), Piñero and Janwa (2014),Hø holdt and Janwa (2012), Hø holdt and Janwal (2009),Janwa and Lal (2003),Janwa and Moreno (1996),Janwa et al. (1995), Dougherty and Janwa (1991), Li (1996), van Lint and Wilson (1992), Lubotzky (1994), Lubotzky et al. (1988), MacWilliams and Sloane (1977), Margulis (1988), Paul et al. (1976/77), Pippenger (1990a),Pippenger and Lin (1994),Pippenger (1990b), Sarnak (1990), Sipser and Spielman (1996), Tanner (1984), Terras (1999)).

For **application of spectral graph theory** to networks (e.g., biomolecular) we suggest Van Mieghem (2011), Masuda et al. (2017), Lovász et al. (1999), Ganguly et al. (2009), Beyer (2009), Solé and Valverde (2004) Piraveenan et al. (2012), Banerjee and Jost (2009), Alderson (2008), Willinger et al. (2009), Chatterjee et al. (2011), Takemoto and Oosawa (2007), Randles et al. (2011)), and general **spectral analysis** applicable to biomolecular networks (Chung (2009), Andersen et al. (2008), Andersen et al. (2007), Chung and Lu (2006), Chung and Lu (2004).

For **algorithmic analysis** of various properties of biomolecular networks discussed below we refer to (Van Mieghem (2011), Masuda et al. (2017), Lovász et al. (1999), Ganguly et al. (2009), Beyer (2009), Solé and Valverde (2004) Piraveenan et al. (2012), Banerjee and Jost (2009), Alderson (2008), Willinger et al. (2009), Chatterjee et al. (2011), Takemoto and Oosawa (2007), Randles et al. (2011)), and general spectral analysis applicable to biomolecular networks (Chung (2009), Andersen et al. (2008), Andersen et al. (2007), Chung and Lu (2006), Chung and Lu (2004)).

REFERENCES

Alderson, D. L. (2008). Catching the "network science" bug: insight and opportunity for the operations researcher. *Oper. Res.* 56, 1047–1065. doi:10.1287/opre.1080.0606

Alon, N. and Spencer, J. H. (1992). *The probabilistic method*. Wiley-Interscience Series in Discrete Mathematics and Optimization (New York: John Wiley & Sons Inc.). With an appendix by Paul Erdős, A Wiley-Interscience Publication

Andersen, R., Chung, F., and Lang, K. (2008). Local partitioning for directed graphs using PageRank. *Internet Math.* 5, 3–22

Andersen, R., Chung, F., and Lu, L. (2007). Drawing power law graphs using a local/global decomposition. *Algorithmica* 47, 379–397. doi:10.1007/s00453-006-0160-2

Babai, L. (1979). Spectra of Cayley graphs. *J. Combin. Theory Ser. B* 27, 180–189. doi:10.1016/0095-8956(79)90079-0

Banerjee, A. and Jost, J. (2009). Graph spectra as a systematic tool in computational biology. *Discrete Appl. Math.* 157, 2425–2431. doi:10.1016/j.dam.2008.06.033

Barg, A. and Zémor, G. (2004). Error exponents of expander codes under linear-complexity decoding. *SIAM J. Discrete Math.* 17, 426–445. doi:10.1137/S0895480102403799

Beyer, A. (2009). Network-based models in molecular biology. In *Dynamics on and of complex networks* (Birkhäuser Boston, Inc., Boston, MA), Model. Simul. Sci. Eng. Technol. 35–56. doi:10.1007/978-0-8176-4751-3_3

Bien, F. (1989). Constructions of telephone networks by group representations. *Notices Amer. Math. Soc.* 36, 5–22

Biggs, N. (1993). *Algebraic graph theory*. Cambridge Mathematical Library (Cambridge: Cambridge University Press), second edn.

Bollobás, B. (1978). *Extremal graph theory*, vol. 11 of *London Mathematical Society Monographs* (Academic Press, Inc. [Harcourt Brace Jovanovich, Publishers], London-New York)

Bollobás, B. (1978). *Extremal graph theory*, vol. 11 of *London Mathematical Society Monographs* (London: Academic Press Inc. [Harcourt Brace Jovanovich Publishers])

Bollobás, B. (1986). *Extremal graph theory with emphasis on probabilistic methods*, vol. 62 of *CBMS Regional Conference Series in Mathematics* (Published for the Conference Board of the Mathematical Sciences, Washington, DC)

Bollobás, B. (1991). Random graphs. In *Probabilistic combinatorics and its applications (San Francisco, CA, 1991)* (Providence, RI: Amer. Math. Soc.), vol. 44 of *Proc. Sympos. Appl. Math.* 1–20

Brouwer, A. E., Cohen, A. M., and Neumaier, A. (1989). *Distance-regular graphs*, vol. 18 of *Ergebnisse der Mathematik und ihrer Grenzgebiete (3) [Results in Mathematics and Related Areas (3)]* (Berlin: Springer-Verlag)

Chatterjee, S., Diaconis, P., and Sly, A. (2011). Random graphs with a given degree sequence. *Ann. Appl. Probab.* 21, 1400–1435. doi:10.1214/10-AAP728

Chung, F. (2009). A local graph partitioning algorithm using heat kernel PageRank. *Internet Math.* 6, 315–330 (2010). doi:10.1080/15427951.2009.10390643

Chung, F. and Lu, L. (2004). The small world phenomenon in hybrid power law graphs. In *Complex networks* (Springer, Berlin), vol. 650 of *Lecture Notes in Phys.* 89–104. doi:10.1007/978-3-540-44485-5_4

Chung, F. and Lu, L. (2006). *Complex graphs and networks*, vol. 107 of *CBMS Regional Conference Series in Mathematics* (Published for the Conference Board of the Mathematical Sciences, Washington, DC; by the American Mathematical Society, Providence, RI). doi:10.1090/cbms/107

Chung, F. R. K. (1966). Can you hear the shape of a graph? *Amer. Math. Monthly*

Chung, F. R. K. (1991). Constructing random-like graphs. In *Probabilistic combinatorics and its applications (San Francisco, CA, 1991)* (Providence, RI: Amer. Math. Soc.), vol. 44 of *Proc. Sympos. Appl. Math.* 21–55

Chung, F. R. K. (1997). *Spectral graph theory*, vol. 92 of *CBMS Regional Conference Series in Mathematics* (Published for the Conference Board of the Mathematical Sciences, Washington, DC; by the American Mathematical Society, Providence, RI)

Chung, F. R. K., Graham, R. L., and Wilson, R. M. (1988). Quasirandom graphs. *Proc. Nat. Acad. Sci. U.S.A.* 85, 969–970. doi:10.1073/pnas.85.4.969

Cvetković, D. M., Doob, M., Gutman, I., and Torgašev, A. (1988). *Recent results in the theory of graph spectra*, vol. 36 of *Annals of Discrete Mathematics* (Amsterdam: North-Holland Publishing Co.)

Cvetković, D. M., Doob, M., and Sachs, H. (1980). *Spectra of graphs*, vol. 87 of *Pure and Applied Mathematics* (New York: Academic Press Inc. [Harcourt Brace Jovanovich Publishers]). Theory and application

Davidoff, G., Sarnak, P., and Valette, A. (2003). *Elementary number theory, group theory, and Ramanujan graphs*, vol. 55 of *London Mathematical Society Student Texts* (Cambridge University Press, Cambridge). doi:10.1017/CBO9780511615825

Delgado, M. and Janwa, H. (2017). On the conjecture on APN functions and absolute irreducibility of polynomials. *Des. Codes Cryptogr.* 82, 617–627. doi:10.1007/s10623-015-0168-1

Diaconis, P. (1988). *Group representations in probability and statistics*. Institute of Mathematical Statistics Lecture Notes—Monograph Series, 11 (Hayward, CA: Institute of Mathematical Statistics)

Diaconis, P. (1991). Finite Fourier methods: access to tools. In *Probabilistic combinatorics and its applications (San Francisco, CA, 1991)* (Providence, RI: Amer. Math. Soc.), vol. 44 of *Proc. Sympos. Appl. Math.* 171–194

Dougherty, R. and Janwa, H. (1991). Covering radius computations for binary cyclic codes. *Math. Comp.* 57, 415–434. doi:10.2307/2938683. With microfiche supplement

Easley, D. and Kleinberg, J. (2010). *Networks, crowds, and markets* (Cambridge University Press, Cambridge). doi:10.1017/CBO9780511761942. Reasoning about a highly connected world

Ganguly, N., Deutsch, A., and Mukherjee, A. (eds.) (2009). *Dynamics on and of complex networks*. Modeling and Simulation in Science, Engineering and Technology (Birkhäuser Boston, Inc., Boston, MA). doi:10.1007/978-0-8176-4751-3. Applications to biology, computer science, and the social sciences

Gleich, D. F. (2015). PageRank beyond the web. *SIAM Rev.* 57, 321–363. doi:10.1137/140976649

Guruswami, V. and Indyk, P. (2001). Expander-based constructions of efficiently decodable codes (extended abstract). In *42nd IEEE Symposium on Foundations of Computer Science (Las Vegas, NV, 2001)* (IEEE Computer Soc., Los Alamitos, CA). 658–667

Hø holdt, T. and Janwa, H. (2012). Eigenvalues and expansion of bipartite graphs. *Des. Codes Cryptogr.* 65, 259–273. doi:10.1007/s10623-011-9598-6

Hø holdt, T. and Janwal, H. (2009). Optimal bipartite Ramanujan graphs from balanced incomplete block designs: their characterizations and applications to expander/LDPC codes. In *Applied algebra, algebraic algorithms, and error-correcting codes* (Springer, Berlin), vol. 5527 of *Lecture Notes in Comput. Sci.* 53–64. doi:10.1007/978-3-642-02181-7_6

Horn, R. A. and Johnson, C. R. (2013). *Matrix analysis* (Cambridge University Press, Cambridge), second edn.

Janwa, H. (2003). Good expander graphs and expander codes: parameters and decoding. In *Applied algebra, algebraic algorithms and error-correcting codes (Toulouse, 2003)* (Springer, Berlin), vol. 2643 of *Lecture Notes in Comput. Sci.* 119–128. doi:10.1007/3-540-44828-4_14

Janwa, H. and Lal, A. K. (2003). On Tanner codes: minimum distance and decoding. *Appl. Algebra Engrg. Comm. Comput.* 13, 335–347. doi:10.1007/s00200-003-0098-4

Janwa, H., McGuire, G. M., and Wilson, R. M. (1995). Double-error-correcting cyclic codes and absolutely irreducible polynomials over $\mathrm{GF}(2)$. *J. Algebra* 178, 665–676. doi:10.1006/jabr.1995.1372

Janwa, H. and Moreno, O. (1996). McEliece public key cryptosystems using algebraic-geometric codes. *Des. Codes Cryptogr.* 8, 293–307. doi:10.1023/A:1027351723034

Janwa, H. and Moreno, O. (1998). Coding-theoretic constructions of some number-theoretic Ramanujan graphs. In *Proceedings of the Twenty-ninth Southeastern International Conference on Combinatorics, Graph Theory and Computing (Boca Raton, FL, 1998)*. vol. 130, 63–76

Janwa, H. and Rangachari, S. (2015). Ramanujan graphs and their applications

Kac, M. (1966). Can one hear the shape of a drum? *Amer. Math. Monthly* 73, 1–23. doi:10.2307/2313748

Levin, D. A. and Peres, Y. (2017). *Markov chains and mixing times* (American Mathematical Society, Providence, RI). Second edition of [MR2466937], With contributions by Elizabeth L. Wilmer, With a chapter on "Coupling from the past" by James G. Propp and David B. Wilson

Li, W. C. W. (1996). *Number theory with applications*, vol. 7 of *Series on University Mathematics* (River Edge, NJ: World Scientific Publishing Co. Inc.)

Lovász, L., Gyárfás, A., Katona, G., Recski, A., and Székely, L. (eds.) (1999). *Graph theory and combinatorial biology*, vol. 7 of *Bolyai Society Mathematical Studies* (János Bolyai Mathematical Society, Budapest)

Lubotzky, A. (1994). *Discrete groups, expanding graphs and invariant measures*, vol. 125 of *Progress in Mathematics* (Basel: Birkhäuser Verlag). With an appendix by Jonathan D. Rogawski

Lubotzky, A. (2012). Expander graphs in pure and applied mathematics. *Bull. Amer. Math. Soc. (N.S.)* 49, 113–162. doi:10.1090/S0273-0979-2011-01359-3

Lubotzky, A., Phillips, R., and Sarnak, P. (1988). Ramanujan graphs. *Combinatorica* 8, 261–277. doi:10.1007/BF02126799

MacWilliams, F. J. and Sloane, N. J. A. (1977). *The theory of error-correcting codes. II* (Amsterdam: North-Holland Publishing Co.). North-Holland Mathematical Library, Vol. 16

Margulis, G. A. (1988). Explicit group-theoretic constructions of combinatorial schemes and their applications in the construction of expanders and concentrators. *Problemy Peredachi Informatsii* 24, 51–60

Masuda, N., Porter, M. A., and Lambiotte, R. (2017). Random walks and diffusion on networks. *Phys. Rep.* 716/717, 1–58. doi:10.1016/j.physrep.2017.07.007

Paul, W. J., Tarjan, R. E., and Celoni, J. R. (1976/77). Space bounds for a game on graphs. *Math. Systems Theory* 10, 239–251. doi:10.1007/BF01683275

Piñero, F. and Janwa, H. (2014). On the subfield subcodes of Hermitian codes. *Des. Codes Cryptogr.* 70, 157–173. doi:10.1007/s10623-012-9736-9

Pippenger, N. (1990a). Communication networks. In *Handbook of theoretical computer science, Vol. A* (Amsterdam: Elsevier). 805–833

Pippenger, N. (1990b). Selection networks. In *Algorithms (Tokyo, 1990)* (Berlin: Springer), vol. 450 of *Lecture Notes in Comput. Sci.* 2–11. doi:10.1007/3-540-52921-7_50

Pippenger, N. and Lin, G. (1994). Fault-tolerant circuit-switching networks. *SIAM J. Discrete Math.* 7, 108–118. doi:10.1137/S0895480192229790

Piraveenan, M., Prokopenko, M., and Zomaya, A. Y. (2012). On congruity of nodes and assortative information content in complex networks. *Netw. Heterog. Media* 7, 441–461. doi:10.3934/nhm.2012.7.441

Randles, M., Lamb, D., Odat, E., and Taleb-Bendiab, A. (2011). Distributed redundancy and robustness in complex systems. *J. Comput. System Sci.* 77, 293–304. doi:10.1016/j.jcss.2010.01.008

Sarnak, P. (1990). *Some applications of modular forms*, vol. 99 of *Cambridge Tracts in Mathematics* (Cambridge: Cambridge University Press). doi:10.1017/CBO9780511895593

Sarnak, P. (2004). What is. . . an expander? *Notices Amer. Math. Soc.* 51, 762–763

Serre, J.-P. (1980). *Trees* (Berlin: Springer-Verlag). Translated from the French by John Stillwell

Sipser, M. and Spielman, D. A. (1996). Expander codes. *IEEE Trans. Inform. Theory* 42, 1710–1722. doi:10.1109/18.556667. Codes and complexity

Solé, R. V. and Valverde, S. (2004). Information theory of complex networks: on evolution and architectural constraints. In *Complex networks* (Springer, Berlin), vol. 650 of *Lecture Notes in Phys.* 189–207. doi:10.1007/978-3-540-44485-5_9

Spielman, D. A. (1996). Linear-time encodable and decodable error-correcting codes. *IEEE Trans. Inform. Theory* 42, 1723–1731. doi:10.1109/18.556668. Codes and complexity

Takemoto, K. and Oosawa, C. (2007). Modeling for evolving biological networks with scale-free connectivity, hierarchical modularity, and disassortativity. *Math. Biosci.* 208, 454–468. doi:10.1016/j.mbs.2006.11.002

Tanner, R. M. (1984). Explicit concentrators from generalized N-gons. *SIAM J. Algebraic Discrete Methods* 5, 287–293. doi:10.1137/0605030

Tanner, R. M. (2001). Minimum-distance bounds by graph analysis. *IEEE Trans. Inform. Theory* 47, 808–821. doi:10.1109/18.910591

Terras, A. (1999). *Fourier analysis on finite groups and applications*, vol. 43 of *London Mathematical Society Student Texts* (Cambridge: Cambridge University Press). doi:10.1017/CBO9780511626265

van Lint, J. H. and Wilson, R. M. (1992). *A course in combinatorics* (Cambridge: Cambridge University Press)

Van Mieghem, P. (2011). *Graph spectra for complex networks* (Cambridge University Press, Cambridge)

Willinger, W., Alderson, D., and Doyle, J. C. (2009). Mathematics and the Internet: a source of enormous confusion and great potential. *Notices Amer. Math. Soc.* 56, 586–599
